# The coarse-grained plaque: a divergent Aβ plaque-type in early-onset Alzheimer’s disease

**DOI:** 10.1007/s00401-020-02198-8

**Published:** 2020-09-14

**Authors:** Baayla D. C. Boon, Marjolein Bulk, Allert J. Jonker, Tjado H. J. Morrema, Emma van den Berg, Marko Popovic, Jochen Walter, Sathish Kumar, Sven J. van der Lee, Henne Holstege, Xiaoyue Zhu, William E. Van Nostrand, Remco Natté, Louise van der Weerd, Femke H. Bouwman, Wilma D. J. van de Berg, Annemieke J. M. Rozemuller, Jeroen J. M. Hoozemans

**Affiliations:** 1 Department of Neurology, Alzheimer Center Amsterdam, Amsterdam Neuroscience, Amsterdam UMC - Location VUmc, Amsterdam, The Netherlands; 2 Department of Pathology, Amsterdam Neuroscience, Amsterdam UMC - Location VUmc, Amsterdam, The Netherlands; 3 Department of Radiology, Leiden University Medical Center, Leiden, The Netherlands; 4 Department of Anatomy and Neurosciences, Amsterdam Neuroscience, Amsterdam UMC - Location VUmc, Amsterdam, The Netherlands; 5 Microscopy and Cytometry Core Facility, Amsterdam Neuroscience, Amsterdam UMC - Location VUmc, Amsterdam, The Netherlands; 6 Department of Neurology, University Hospital Bonn, University of Bonn, Bonn, Germany; 7 Department of Clinical Genetics, Amsterdam Neuroscience, Amsterdam UMC - Location VUmc, Amsterdam, The Netherlands; 8 Department of Biomedical and Pharmaceutical Sciences, George & Anne Ryan Institute for Neuroscience, University of Rhode Island, Kingston, USA; 9 Department of Pathology, Leiden University Medical Center, Leiden, The Netherlands; 10 Department of Human Genetics, Leiden University Medical Center, Leiden, The Netherlands

**Keywords:** Amyloid-beta, Coarse-grained plaque, Early-onset Alzheimer’s disease, Cerebral amyloid angiopathy, Neuroinflammation

## Abstract

Alzheimer’s disease (AD) is characterized by amyloid-beta (Aβ) deposits, which come in myriad morphologies with varying clinical relevance. Previously, we observed an atypical Aβ deposit, referred to as the coarse-grained plaque. In this study, we evaluate the plaque’s association with clinical disease and perform in-depth immunohistochemical and morphological characterization. The coarse-grained plaque, a relatively large (Ø ≈ 80 µm) deposit, characterized as having multiple cores and Aβ-devoid pores, was prominent in the neocortex. The plaque was semi-quantitatively scored in the middle frontal gyrus of Aβ-positive cases (*n* = 74), including non-demented cases (*n* = 15), early-onset (EO)AD (*n* = 38), and late-onset (LO)AD cases (*n* = 21). The coarse-grained plaque was only observed in cases with clinical dementia and more frequently present in EOAD compared to LOAD. This plaque was associated with a homozygous *APOE* ε4 status and cerebral amyloid angiopathy (CAA). In-depth characterization was done by studying the coarse-grained plaque’s neuritic component (pTau, APP, PrP^C^), Aβ isoform composition (Aβ_40_, Aβ_42_, Aβ_N3pE_, pSer8Aβ), its neuroinflammatory component (C4b, CD68, MHC-II, GFAP), and its vascular attribution (laminin, collagen IV, norrin). The plaque was compared to the classic cored plaque, cotton wool plaque, and CAA. Similar to CAA but different from classic cored plaques, the coarse-grained plaque was predominantly composed of Aβ_40_. Furthermore, the coarse-grained plaque was distinctly associated with both intense neuroinflammation and vascular (capillary) pathology. Confocal laser scanning microscopy (CLSM) and 3D analysis revealed for most coarse-grained plaques a particular Aβ_40_ shell structure and a direct relation with vessels. Based on its morphological and biochemical characteristics, we conclude that the coarse-grained plaque is a divergent Aβ plaque-type associated with EOAD. Differences in Aβ processing and aggregation, neuroinflammatory response, and vascular clearance may presumably underlie the difference between coarse-grained plaques and other Aβ deposits. Disentangling specific Aβ deposits between AD subgroups may be important in the search for disease-mechanistic-based therapies.

**Electronic supplementary material:**

The online version of this article (10.1007/s00401-020-02198-8) contains supplementary material, which is available to authorized users.

## Introduction

Amyloid-beta (Aβ) plaques and hyperphosphorylated tau (pTau) tangles are the main pathological hallmarks of Alzheimer’s disease (AD). Aβ plaques originate from the accumulation and aggregation of the Aβ peptide, which is formed by the sequential cleavage of the amyloid precursor protein by β- and γ-secretases [21]. Depending on the cleavage site of γ-secretase, the Aβ peptide length may vary from 36 to 43 amino acids, with Aβ_40_ and Aβ_42_ being the most common forms found in AD. The longer Aβ_42_ is more prone to aggregate and is predominantly found in parenchymal plaques, whereas the shorter Aβ_40_ is secreted by cells in higher levels and is the major Aβ isoform deposited in the cerebral vasculature, referred to as cerebral amyloid angiopathy (CAA) [5, 6, 28]. The Aβ peptide can undergo several post-translational modifications resulting, e.g., in truncated pyroglutamate Aβ (Aβ_N3pE_) and Aβ phosphorylated at serine 8 (pSer8Aβ) [30–32]. These different Aβ isoforms are associated with clinical disease progression and are considered to mark sequential phases of plaque and CAA maturation [20, 43, 52].

Since the first description of AD, many different types of amyloid deposits have been described, which vary in their association with clinical symptoms [18, 19, 39, 48, 49]. The currently used categorization scheme for both cerebral parenchymal and vascular Aβ deposits, referred to as plaques and CAA, respectively, are both described by Thal et al. [48, 49]. The first categorization step for plaques is based on their fibril content, since the fibrillar type is better associated with clinical dementia than the non-fibrillar type [11, 44]. Fibrillar plaques can range in size from 30 to 100 μm cross-sectionally and contain Aβ in a β-pleated sheet secondary conformation, also referred to as amyloid. Amyloid plaques are further ordered into compact or cored-only plaques, and classic cored plaques [16, 48]. Non-fibrillar or better known as diffuse plaques, can range from 10 to > 100 μm cross-sectionally with a great variation in morphology [48]. Contrary to fibrillar plaques, diffuse plaques are often present in cases without cognitive impairment [3, 12, 40]. The next categorization step is the presence of a neuritic component. A plaque is considered neuritic when the focal Aβ deposit contains degenerating axons and dendrites referred to as dystrophic neurites. The load of these neuritic plaques in the neocortex correlates well with clinical disease severity [34]. Besides the common plaque variants, more rare plaque types such as the cotton wool plaque are described. These plaques are clinically relevant as, although reported in a few sporadic cases, they are predominantly described in patients with specific *PSEN1* mutations [10, 47]. For vascular Aβ deposits, CAA is categorized as either CAA-Type 1 involving cortical capillaries in additional to leptomeningeal and cortical arteries and arterioles, and CAA-Type 2 not involving cortical capillaries [49].

We recently observed a plaque morphology that did not fit the afore mentioned descriptions [7]. Comparing AD related pathology in a small cohort of different AD subtypes, we observed a relatively large plaque with a coarse-grained structure using anti-Aβ immunostaining. According to our knowledge, these coarse-grained plaques have not yet been well-described. In the current study, we describe the coarse-grained plaque and investigate its presence in a cohort (*n* = 74) of EOAD, LOAD, and Aβ-positive non-demented cases. We further characterize the coarse-grained plaque by comparing it to other clinically relevant Aβ deposits, being the classic cored plaque, the cotton wool plaque, and CAA-Type 1. Confocal laser scanning microscopy (CLSM) was used to image the coarse-grained plaque in 3D.

## Methods

### Post-mortem brain tissue

Post-mortem brain tissue was obtained from the Netherlands Brain Bank (NBB; Amsterdam, The Netherlands, https://www.brainbank.nl) and the Normal Aging Brain Collection (NABCA; Amsterdam UMC - location VUmc, Amsterdam, The Netherlands, http://nabca.eu). In compliance with all ethical standards, brain donors signed informed consent regarding the usage of their brain tissue and clinical records for research purposes. The local medical ethics committee of the VUmc approved the brain donor programs of the NBB and NABCA. Brain dissection and neuropathological diagnosis were performed according to international guidelines of Brain Net Europe II (BNE) consortium (http://www.brainnet-europe.org) and NIA-AA [35]. Aβ-positive cases were selected if cognitive decline was not reported during life, Aβ deposits were present in the brain, i.e., Aβ-positive, and total AD neuropathologic score according to the NIA-AA was ‘low’ [35]. Aβ-positive cases were age- and sex-matched to late-onset AD (LOAD) cases. AD cases were selected based on dementia diagnosis during life in combination with an intermediate or high score for AD pathology [35]. This resulted in a cohort of 15 Aβ-positive cases, 38 early-onset AD (EOAD) -, and 21 LOAD cases. Additional cases, containing cotton wool plaques (*n* = 4) and CAA-Type 1 (*n* = 3) were included for the comparison to different types of Aβ deposits. For all cases, Thal phase for Aβ, Braak stage for neurofibrillary tangles [8], Thal stage for CAA [50] and concomitant pathologies, i.e., Lewy bodies [1], TDP43 pathology [36], and vascular lesions were reported when the respective assessment was readily available from the brain bank (see Supplementary Material 1, Table S1, Online Resource, for case details).

Formalin (4%)-fixed paraffin-embedded (FFPE) tissue blocks of the right middle frontal gyrus were used. In addition, for 14 cases (Supplementary Material 1, Table S1, Online Resource) also FFPE blocks of the right temporal -, parietal -, occipital-, olfactory cortex, pre- and postcentral gyrus, amygdala, hippocampus (including CA1-CA4, dentate gyrus, subiculum, and entorhinal cortex), caudate nucleus, putamen, substantia nigra, locus coeruleus, pons, medulla oblongata, and cerebellum were used. For 1 of 4 cotton wool cases, the superior parietal lobule region was included due to tissue region availability within the NBB. For CAA-Type 1 comparison, the occipital cortex was used, as this region is most prone to CAA pathology [6]. FFPE tissue was cut at 6 μm thickness for immunohistochemistry and at 5 μm thickness for multi-label immunofluorescence. Formalin-fixed free-floating (FFFF) (4% formalin; for 24–36 h) tissue from the left middle frontal gyrus of 5 AD cases (Supplementary Material 1, Table S1, Online Resource) was used for 3D multi-label immunofluorescence confocal imaging. FFFF tissue was put on sucrose (15%; 30%) for cryopreservation, stored at − 80 °C and cut on a sliding microtome in 60 μm-thick sections.

### Genotyping

DNA was extracted from blood or brain tissue and apolipoprotein genotype (*APOE*) was determined for 72 of 74 cases and 3 of 4 cotton wool cases using methods previously described (Supplementary Material 1, Table S1, Online Resource) [54]. Whole-exome sequencing (WES) was performed for 64 of 74 cases and for 3 of 4 cotton wool cases. For WES, exome-DNA was captured using the SeqCap EZ Human Exome Library v3.0 capture Kit or the Agilent SureSelect V6 kit (58 M); 150 bp paired-end reads were generated on the Illumina platform, with a mean depth of coverage of at least 30 × across exons. We performed in house alignment and quality control. The exomes of the genes *APP* (NM_000484.3), *PSEN1* (NM_000021.3), and *PSEN2* (NM_000447.2) were analyzed for likely pathogenic (class 4) or pathogenic (class 5) variants according to variant classification consensus guidelines [42]. Known pathogenic mutations are reported (Supplementary Material 1, Table S1, Online Resource).

### (Immuno)histochemistry

(Immuno)histochemistry was performed on sequential sections for hematoxylin and eosin (H&E), Congo red, Aβ (aa 8-17), Aβ_40_, Aβ_42_, Aβ_N3pE_, pSer8Aβ, APP, PrP^C^, pTau, C4b, MHC-II, GFAP, norrin, laminin, collagen IV, and ApoE (see Supplementary Material 1, Table S2 for antibody—and staining specifics, Online Resource). After deparaffinization, (immuno)histochemistry was performed according to the following protocols. H&E was performed by submerging sections in hematoxylin (5 min), followed by submerging in eosin (3 min). Histochemistry for Congo red was performed by incubation with a saturated sodium chloride solution (3% NaCl in 80% ethanol and 1% 1 M NaOH) followed by incubation with a saturated Congo red solution (0.25% Congo red in 80% ethanol and 1% 1 M NaOH), both for 20 min at room temperature. Endogenous peroxidase was quenched using 0.3% H_2_O_2_ in either phosphate buffered saline (PBS; pH 7.4) or methanol. This was followed by the appropriate antigen retrieval and primary antibody incubation. Subsequently, sections were incubated with a secondary antibody, followed by color development using 3,3’-diaminobenzidine (DAB) (Agilent-Dako) and counterstained using haematoxylin. In between steps, sections were rinsed in PBS. Finally, sections were mounted with Quick-D (Klinipath) or Entellan (Sigma-Aldrich) and coverslipped. The omission of primary antibodies was used as negative control.

### Multi-label immunofluorescence

Multi-labeling on 5 µm sections was performed on case #18, 32, 35, 39, 56, 64, 68, and all cotton wool cases (#75, 76, 77, 78) for neuroinflammatory markers and vascular-associated markers. Double-labeling for Aβ_40_ and Aβ_42_ was in addition to the above-mentioned cases, also performed on case #79, 80, 81. See Supplementary Material 1, Table S2, Online Resource, for antibody and staining details. After deparaffinization, sections stained for markers reflecting neuroinflammation were heated in citrate buffer (pH 6.0) using an autoclave and subsequently incubated overnight with a cocktail of antibodies directed against C4b, CD68, and GFAP. The following day, sections were incubated for 1 h with a cocktail of the appropriate secondary antibodies (dilution 1:250). Antibody retrieval for sections stained for vascular-associated markers entailed submerging sections in Tris–EDTA buffer (pH 9.0) and heating them using an autoclave. Primary antibody incubation was done sequentially, since both primary antibodies for vascular-associated markers were raised in rabbit. First, sections were incubated with anti-laminin overnight, followed by incubation with EnVision (Dako), rinsed in Tris-buffered saline (TBS pH 7.6) and color development by tyramide reagent Alexa 568 (1:100 in 0.0015% H_2_O_2_ in TBS). Sections were heated in Tris–EDTA buffer using a microwave to ensure primary antibody detachment. Subsequently, sections were incubated with anti-norrin and anti-Aβ aa 1-16 directly labeled Alexa 488. Norrin was visualized by incubating sections for 1 h with secondary antibody goat-anti-rabbit Alexa 647 (dilution 1:250). Multi-label staining for Aβ isoform staining was performed by submerging sections in 98% formic acid for 5 min, followed by incubation with a cocktail of primary antibodies overnight and secondary antibodies the next day for 1 h. All sections were enclosed with 80% glycerol/20% TBS. In between steps, sections were rinsed in PBS. All antibodies were diluted in normal antibody dilution (ImmunoLogic).

Multi-labeling on 60 µm FFFF sections was performed on case #29, 30, 42, 53, and 74. Sections were stained for neuroinflammatory markers (CD68 and GFAP together with Aβ aa 1-16), vascular markers (norrin and laminin together with Aβ aa 1-16), or Aβ_40_ together with Aβ_42_ (see Supplementary Material 1, Table S2, Online Resource). Antigen retrieval was performed for neuroinflammatory and vascular markers by heating sections in a water bath to 95 °C for 30 min in either citrate buffer (pH 6.0) or Tris–EDTA buffer (pH 9.0). Sections double-labeled for Aβ_40_ and Aβ_42_ were submerged in formic acid 99% for 10 min. All sections were rinsed in 2% bovine serum albumin (BSA) in TBS to block non-specific binding sites in the tissue sample and prevent non-specific antibody binding. Since some primary antibodies were raised in the same species, staining for neuroinflammatory and vascular markers was performed for each antigen sequentially using consecutive incubation steps. After the first primary antibody incubation with either anti-CD68 or anti-laminin, sections were incubated with a biotinylated secondary antibody directed against the appropriate species. This was followed by the ABC method and color development using tyramide reagent (Alexa 594 for CD68; Alexa 555 for laminin). Sections were re-heated in the water bath to 95 °C for 30 min to ensure the detachment of the primary antibody. The potential residual primary antibody sites were then blocked by incubation for 1 h with normal serum (2%) of the appropriate species. This was followed by incubation with the next primary antibody (anti-GFAP or anti-norrin) and secondary antibody (goat-anti-chicken Alexa 555 or goat-anti-rabbit Alexa 647). The staining procedure was finalized by incubation with anti-Aβ aa 1-16 directly labeled Alexa 488. Staining for Aβ_40_ and Aβ_42_ was performed using a cocktail of antibodies and visualized with goat-anti-mouse-IgG2 Alexa 488 and goat-anti-mouse-IgG1 Alexa 647. All sections were mounted with 0.3% gelatin in Tris–HCl (0.05 M; pH 7.6), enclosed with Mowiol and DABCO and coverslipped. In between steps, sections were rinsed in TBS. For all multi-labeling experiments, the omission of primary antibodies was used as a negative control and the color development step with tyramide was performed after the detachment step to check if primary antibody detachment was successful.

### Confocal imaging

CLSM was performed with a Nikon A1R HD microscope (Nikon, Amsterdam, the Netherlands) using a CFI Plan Apochromat λ 100x oil, NA 1.45, WD 0.13 objective lens. Scanning was done using galvano with scan size 1024 × 1024 pixels, pixel size: 0.12 μm, and a pixel dwell time of 1.1 μs. Signal detection was performed using an A1-DUS spectral detector unit. Pinhole size was adjusted per experiment. The pinhole size was set to 57.5 μm for all IF stained FFPE sections (5 μm). For FFFF sections (60 μm), the pinhole radius was set to 20.4 μm for Aβ_40_ and Aβ_42_ combination, to 66.4 μm for neuroinflammatory marker combination (Aβ, CD68, and GFAP), and to 63.9 μm for vascular marker combination (Aβ, laminin, and norrin). Sections were irradiated with a laser combination of wavelength 488, 514, 594, and 640 nm, depending on fluorochrome combination. FFPE sections (5 μm) were imaged in 2D. For a 3D reconstruction of coarse-grained plaques, Z-steps of 0.25 μm were taken during the scanning of FFFF sections. For FFPE sections, no line integration was performed. For FFFF sections, line integration was set to 8 × for neuroinflammatory marker stained sections, to 4 × for vascular marker stained sections, and to 4 × for Aβ_40_ and Aβ_42_ stained sections. The spectrum profile of each fluorochrome was acquired from single stains. The autofluorescent spectrum was acquired from negative control sections. Images were spectrally unmixed using the appropriate spectra in the NIS-Elements AR software (Nikon).

### 3D Image processing and qualitative analysis of coarse-grained plaques

Coarse-grained plaques were selected and subsequently scanned based on their 2D morphology in Aβ aa 1-16, Aβ_40_, or norrin staining with the CSLM. In total, 118 plaques were qualitatively analyzed in 5 cases (Supplementary Material 1, Table S3, Online Resource). The GFAP—Alexa 555 signal that was used for the neuroinflammation protocol in FFFF sections, decreased along the *z* axis due to spherical aberration. This effect was post-imaging corrected in Imaris software (version 9.3.1, Oxford Instruments) by normalizing all layers. Post-imaging processing was performed for all FFFF z-stacks using the denoise.AI algorithm in NIS-Elements imaging software (version 5.20.01, Nikon). Movies were annotated using Adobe After Effects (version 16.1, Adobe Systems Incorporated). All figures were composed using Adobe Photoshop (CS6, Adobe Systems Incorporated).

### Semi-quantitative scoring of the coarse-grained plaque

In anti-Aβ immunohistochemistry, the coarse-grained plaque is defined by its size (30-100 µm), coarse-grainy deposits with multiple intensely stained cores, Aβ-devoid pores, and an ill-defined border (see “Results” section). Its presence was semi-quantitatively scored independently by two assessors (BDCB, MB) after anti-Aβ (aa 8-17; 6F/3D) staining. The assessors were blinded for group (Aβ-positive cases, EOAD, or LOAD) during the scoring process. Interrater reliability for the semi-quantitative scoring of the coarse-grained plaque was substantial (Cohen’s *κ* = 0.78) [9]. The scoring system was comprised of the following 4 categories: 0, no coarse-grained plaques per 1 cm^2^; 1, sparse (> 0 and < 6 coarse-grained plaques per 1 cm^2^); 2, moderate (≥ 6 and ≤ 30 coarse-grained plaques per 1 cm^2^); and 3, frequent (> 30 coarse-grained plaques per 1 cm^2^). See Supplementary Material 1, Fig. S4, Online Resource, for representative figures per category.

### Laser-capture microdissection of coarse-grained plaques for ELISA analysis

Snap-frozen brain tissue of 2 cases with a frequent score for coarse-grained plaques (#18 and 32) was sectioned at 25 µm and mounted on Leica Frame Slides (Leica Microsystems, Danvers, MA, USA). Mounted brain sections were stained with thioflavin S to identify fibrillar amyloid plaques. Per case, a total of 1200 individual coarse-grained amyloid plaques were identified, excised, and captured using a laser-capture microdissection microscope LMD6 (Leica Microsystems) (See Supplementary Material 1, Fig. S5, Online Resource, for visualization of plaque laser microdissection). The microdissected plaques were collected into 100 µl of 10 mM sodium phosphate buffer, pH 7.2, containing 0.01% sodium azide.

### Aβ ELISA analysis of captured coarse-grained plaques

For the analysis of Aβ composition, 10 µl of the collected plaque suspension was added to 90 µl 5 M Guanidine HCl, 50 mM Tris–HCl, pH 8.0. The samples were then diluted 1:200 and subjected to sandwich ELISA analysis for the measurement of Aβ_40_ and Aβ_42_ peptides as described [13, 29]. Briefly, in the sandwich ELISAs Aβ_40_ and Aβ_42_ were captured using their respective carboxyl-terminal specific antibodies mAb2G3 and mAb21F12 and biotinylated m3D6, specific for N-terminus of human Aβ, was used for detection [29]. Each plaque collection sample was measured in triplicate and compared to linear standard curves generated with known concentrations of human Aβ_40_ and Aβ_42_ using a Spectramax M2 plate reader (Molecular Devices, Sunnyvale, CA).

### Statistical analysis

Demographics of Aβ-positive cases, EOAD, and LOAD were compared using Chi square test for categorical data and ANOVA for numerical data. Since Aβ-positive cases were age- and sex- matched to LOAD, age and sex comparison was only performed for EOAD versus LOAD. Chi square test was performed to study the presence of coarse-grained plaques in relation to group (Aβ-positive cases, EOAD, and LOAD), *APOE* ε4 status (non-carrier, ε4 heterozygous, and ε4 homozygous), and CAA-Type (no CAA, CAA-Type 1, and CAA-Type 2). Post hoc Chi square test was performed when initial test was significant. A *p* value of < 0.05 was considered significant. Statistical analysis was performed in IBM SPSS version 22.0.

## Results

### Description and localization of the coarse-grained plaque

**Fig. 1:**
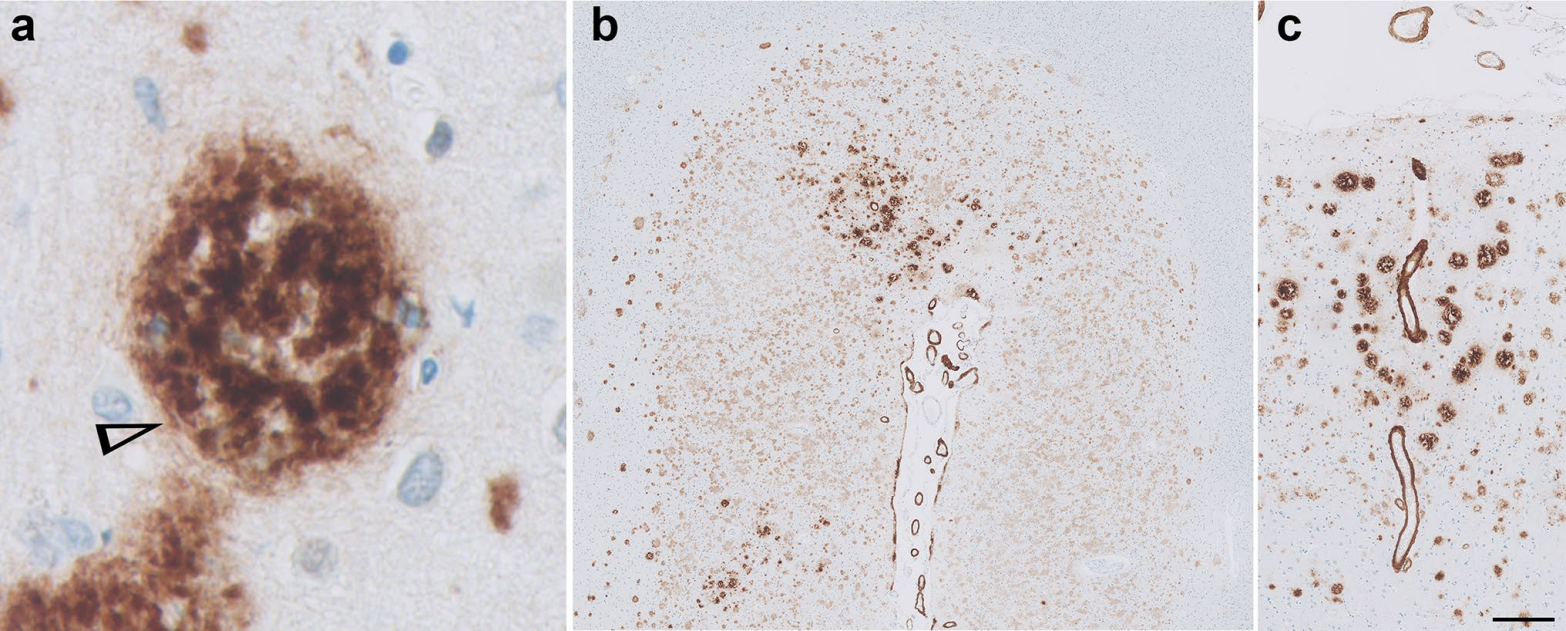
The coarse-grained plaque and its localization. **a** The coarse-grained plaque was defined using Aβ immunohistochemistry (aa 8-17; 6F/3D) by its size (30–100 µm), coarse-grainy deposits with the appearance of multiple cores, Aβ-devoid pores, and a vague rim. Tubular-like structures (arrowhead) were seen within the plaque. **b** The coarse-grained plaque was predominantly found in layers II–IV of sulcal fundi and **c** near CAA-affected vessels. Scale bar is applicable to all images and represents 10 µm in **a**, 400 µm in **b**, and 200 µm in **c**. *Aβ* amyloid-beta, *CAA* cerebral amyloid angiopathy

Morphologically the coarse-grained plaque showed a multi-cored, coarse-grainy appearance with pores devoid of Aβ (Fig. 1a). The coarse-grained plaque is usually relatively large, with a diameter (Ø) ranging between 50 and 100 µm. However, smaller variants (Ø ≈ 30 µm) were also observed. Tubular-like or trabecular structures were seen within the plaque. Immunostaining directed against Aβ (aa 8-17; 6F/3D) revealed a somewhat ill-defined plaque border (Figs. 1a, 3a). The plaques were predominantly found in layers II–IV, but when frequently present also in layers V and VI. The coarse-grained plaques were mostly observed in clusters in the fundi of cortical sulci (Fig. 1b) and near CAA-affected vessels (Fig. 1c). In summary, using anti-Aβ immunohistochemistry the coarse-grained plaque can be distinguished by its size (30-100 µm), coarse-grainy deposits with the appearance of multiple cores, Aβ-devoid pores, and a vague rim. In a subset of cases (*n* = 14) the plaque’s presence was scored in 20 brain regions additional to the middle frontal gyrus (Supplementary Material 1, Fig. S6, Online Resource). The coarse-grained plaque was predominantly found in the frontal and parietal regions and to a lesser extend also in the temporal and occipital regions of the neocortex. The coarse-grained plaque was moderately observed in the limbic regions, being mostly the olfactory cortex and amygdala and occasionally the hippocampus. The coarse-grained plaque was not observed in the basal ganglia. In one case, a few coarse-grained plaques were observed in the olivary nuclei and in another case one coarse-grained plaque was seen in the cerebellum. Other cases did not show coarse-grained plaques in the brain stem areas or cerebellum.

## Clinical relevance of the coarse-grained plaque

### Cohort description

**Fig. 2:**
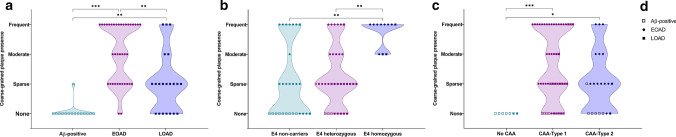
Clinical relevance of coarse-grained plaques. **a** Coarse-grained plaques were generally not observed in Aβ-positive non-demented cases, except for 1 coarse-grained plaque in 1 case. Coarse-grained plaques were more frequently seen in EOAD compared to LOAD. **b** The plaque’s presence was related to a homozygous *APOE* ε4 status. *APOE* status was known for 72 of 74 cases. **c** The coarse-grained plaque was not observed in cases devoid of CAA. **d** Figure legend is applicable to all 3 previous graphs. *Aβ* amyloid-beta, *CAA* cerebral amyloid angiopathy, *EOAD* early-onset Alzheimer’s disease, *LOAD* late-onset Alzheimer’s disease. Statistical analyses was performed using (post hoc) Chi square test; *p < 0.05; **p < 0.01; ***p < 0.001

We investigated the clinical relevance of the coarse-grained plaque by scoring the plaque in a cohort including EOAD and LOAD cases as well as non-demented Aβ-positive cases (see Supplementary Material 1, Table S1, Online Resource, for individual case details; Table 1 for group demographics; Fig. 2a for coarse-grained plaque scoring results per group). The EOAD group differed from the Aβ-positive group in their age at death and, as per definition, disease duration and AD pathology. EOAD cases had higher Braak stages for neurofibrillary tangles compared to LOAD cases (*p* < 0.01). Parenchymal CAA was seen in 66% of Aβ-positive cases, in 100% of EOAD, and in 91% of LOAD. Of the Aβ-positive cases that were tested for *APOE*, only 31% had an ε4 allele and all ε4 carriers were heterozygous. In the EOAD group 58% of cases was ε4 positive compared to 86% of LOAD cases. The EOAD ε4 positive group consisted of relatively more ε4 homozygous cases (36%) than the LOAD ε4 group (17%). In 5 cases of the EOAD group an autosomal dominant mutation was found in either the *PSEN1* (*n* = 4) or *APP* gene (n = 1) (Supplementary Material 1, Table S1, Online Resource).

**Table 1:** Group demographics

	Aβ-positive cases	EOAD	LOAD
	*n* = 15	*n* = 38	*n* = 21
Male, *n* (%)	8 (53)	25 (66)	9 (43)
Age of onset	NA	55 (± 8)***	76 (± 6)***
Disease duration	NA	10 (± 6)***	8 (± 3)
Age at death	80 (± 9)	65 (± 9)***	85 (± 6)***
ABC [35]			
A: *n* per stage 0/1/2/3	0/13/2/0	0/0/0/38***	0/0/0/21
B: *n* per stage 0/1/2/3	1/11/3/0	0/0/0/38***	0/0/4/17**
C: *n* per stage 0/1/2/3	11/4/0/0	0/0/3/35***	0/2/4/15
CAA-Type [49]			
NA/1/2	5/4/6	0/25/13***	2/17/2*
*APOE* ε4 allele			
*n* per non-carrier/heterozygous/homozygous	9/4/0	16/14/8	3/15/3*

Data are mean ± SD; age at onset, disease duration, and age at death shown in years; Aβ-positive cases were age- and sex-matched to LOAD. Statistical analysis was performed for group differences between Aβ-positive cases versus EOAD and EOAD versus LOAD using ANOVA for continuous data and (post hoc) Chi square test for categorical data

*Aβ* amyloid-beta, *EOAD* early-onset Alzheimer’s disease, *LOAD* late-onset Alzheimer’s disease, *n* number, *NA* non-applicable, *SD* standard deviation

*p < 0.05; **p < 0.01; ***p < 0.001

### The coarse-grained plaque is associated with an early disease onset and a homozygous *APOE* ε4 status

Coarse-grained plaques were not observed in the non-demented Aβ-positive cases (with the exception of 1 coarse-grained plaque in the middle frontal gyrus of case #7). In contrast, the plaque was present in 66% of LOAD and in 95% of EOAD patients (Fig. 2a). Extensive genotyping for (likely) pathogenic mutations in the *APP*, *PSEN1*, and *PSEN2* genes was performed for 64 cases. In 5 genotyped cases with such a mutation, coarse-grained plaques were observed in either a sparse (*n* = 2), moderate (*n* = 1) or frequent (*n* = 2) amount (Supplementary Material 1, Table S1, Online Resource). Although coarse-grained plaques were observed in both ε4 carriers and ε4 non-carrier AD cases, the plaque was especially prevalent in individuals homozygous for the ε4 allele (*p* < 0.01) (Fig. 2b). Moreover, homozygous carriers of the ε4 allele (*n* = 11; 3 LOAD, 8 EOAD), always showed a moderate to frequent amount of coarse-grained plaques.

#### The coarse-grained plaque is associated with CAA-Type 1

Due to our previous observation that coarse-grained plaques often neighboured CAA-affected vessels, we investigated the co-occurrence of coarse-grained plaques with the presence and type of CAA [49] (Fig. 2c). Note that only 7 cases were devoid of CAA, of which 2 were cases with clinical AD. The coarse-grained plaque was only observed in cases with CAA. A frequent degree of coarse-grained plaques was observed in 47% of cases with CAA-Type 1 and in 14% of cases with CAA-Type 2. The presence of CAA-Type 1 was correlated to the presence of coarse-grained plaques (*p* < 0.001).

## In-depth characterization of the coarse-grained plaque

### Coarse-grained plaques are different from cotton wool and classic cored plaques

**Fig. 3:**
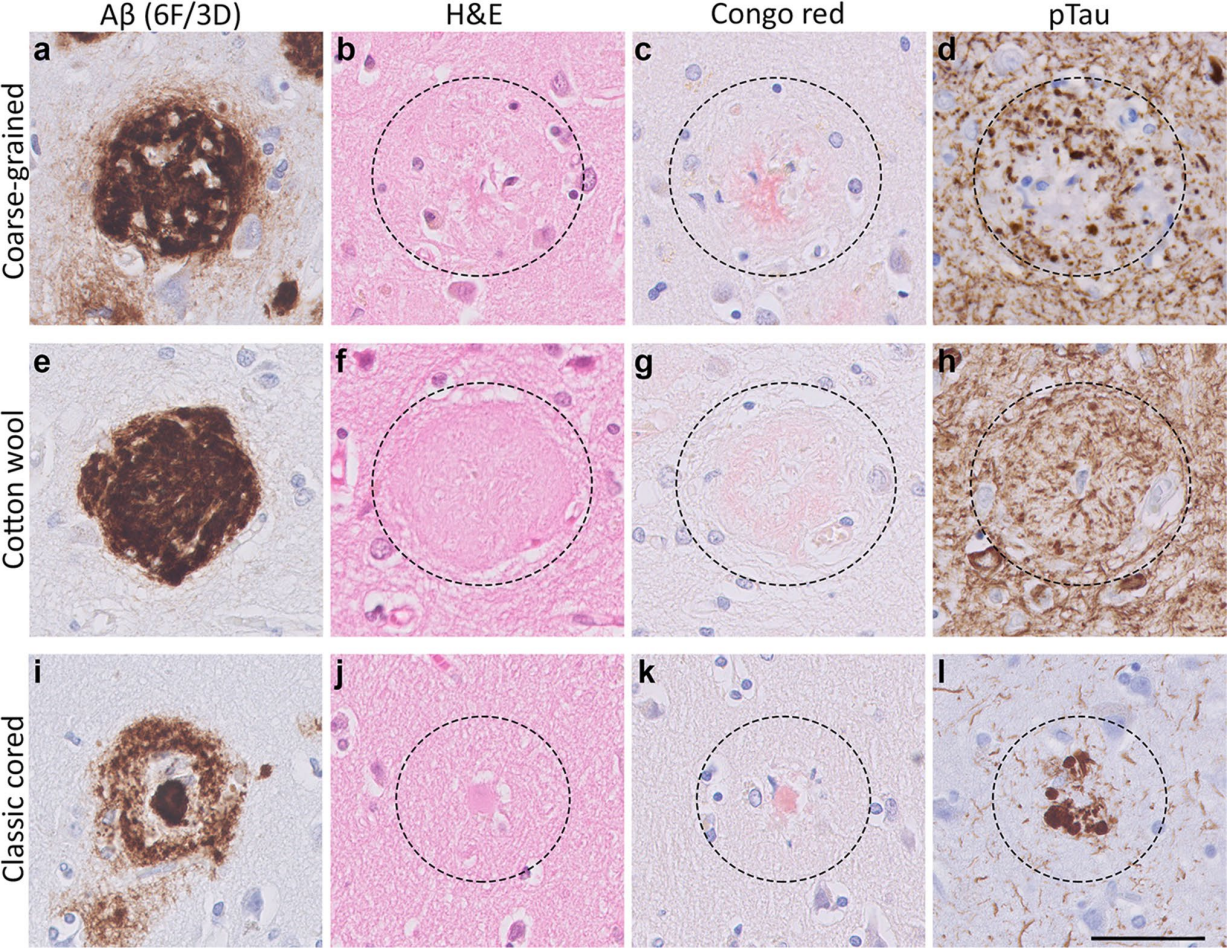
Histology of the coarse-grained plaque compared to the cotton wool and classic cored plaque. **a** Anti-Aβ (6F/3D) staining showed that the coarse-grained plaque is relatively large (⌀ ≈ 80 µm), contains Aβ-devoid pores, and has an ill-defined border. **b** The coarse-grained plaque showed tissue distortion in H&E. **c** Congo red staining for the coarse-grained plaque demonstrated fibrillar amyloid not condensed in one core. **d** When the coarse-grained plaque contained pTau immunoreactive dystrophic neurites, the plaque-center was often devoid of pTau immunoreactivity. **e**, **f** The cotton wool plaque showed a distinct circumscribed border in both anti-Aβ (6F/3D) as well as H&E staining. **g** The cotton wool plaque lacked clear amyloid. **h** Neuritic threads indicated by pTau immunoreactivity, but not dystrophic neurites were seen within the cotton wool plaque. **i** The classic cored plaque demonstrated a central amyloid core, which was surrounded by a corona of non-fibrillar Aβ. **j**–**l** Central pit in classic cored plaques was visible in H&E as well as Congo red staining and was often surrounded by dystrophic neurites (pTau). Scale bar represents 50 µm and is applicable to all images. *Aβ* Amyloid-beta, *H&E* hematoxylin–eosin, *pTau* hyperphosphorylated tau

**Fig. 4:**
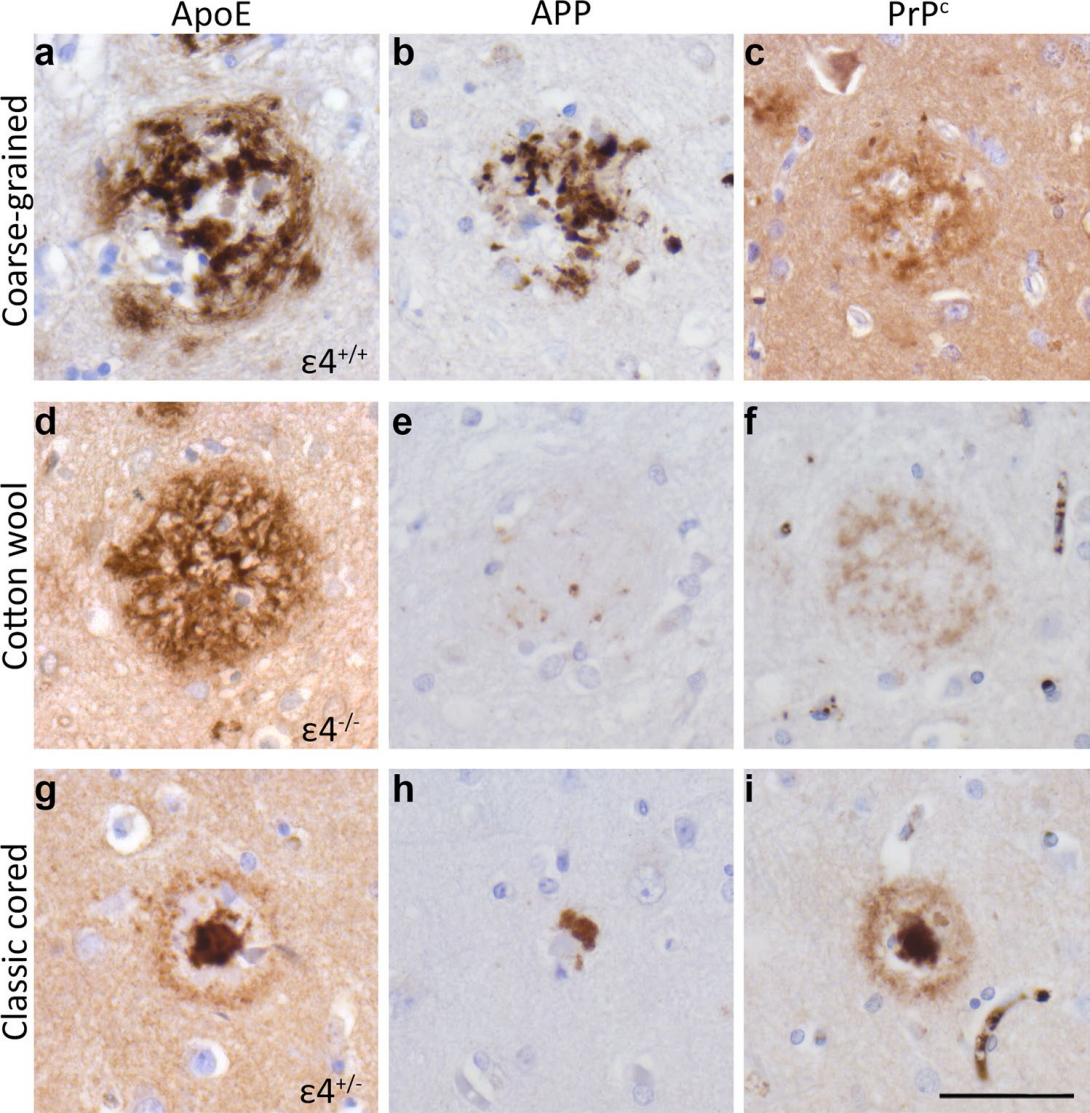
ApoE, APP, and PrP^C^ immunoreactivity of the coarse-grained plaque compared to the cotton wool and classic cored plaque. **a** ApoE was abundantly present all through the coarse-grained plaque. **b** Dystrophic neurites immunoreactive for APP were found throughout the coarse-grained plaque. **c** The coarse-grained plaque was immunoreactive for PrP^C^. **d** The cotton wool plaque stained positive for ApoE. **e** APP dystrophic neurites were negligibly visible in the cotton wool plaques. **f** PrP^C^ was observed in cotton wool plaques. **g** ApoE was found in both the corona and core of the classic cored plaque. ApoE staining intensity was highest in the core. **h** APP dystrophic neurites could be seen surrounding the core of classic cored plaques. **i** The classic cored plaque was immunoreactive for PrP^C^. *APOE* genotype of the respective case is shown in right lower corner of images of plaques stained for ApoE. Scale bar represents 50 µm and is applicable to all images. *ApoE* apolipoprotein E, *APP* amyloid precursor protein, *PrP*^*C*^ cellular prion protein

**Fig. 5:**
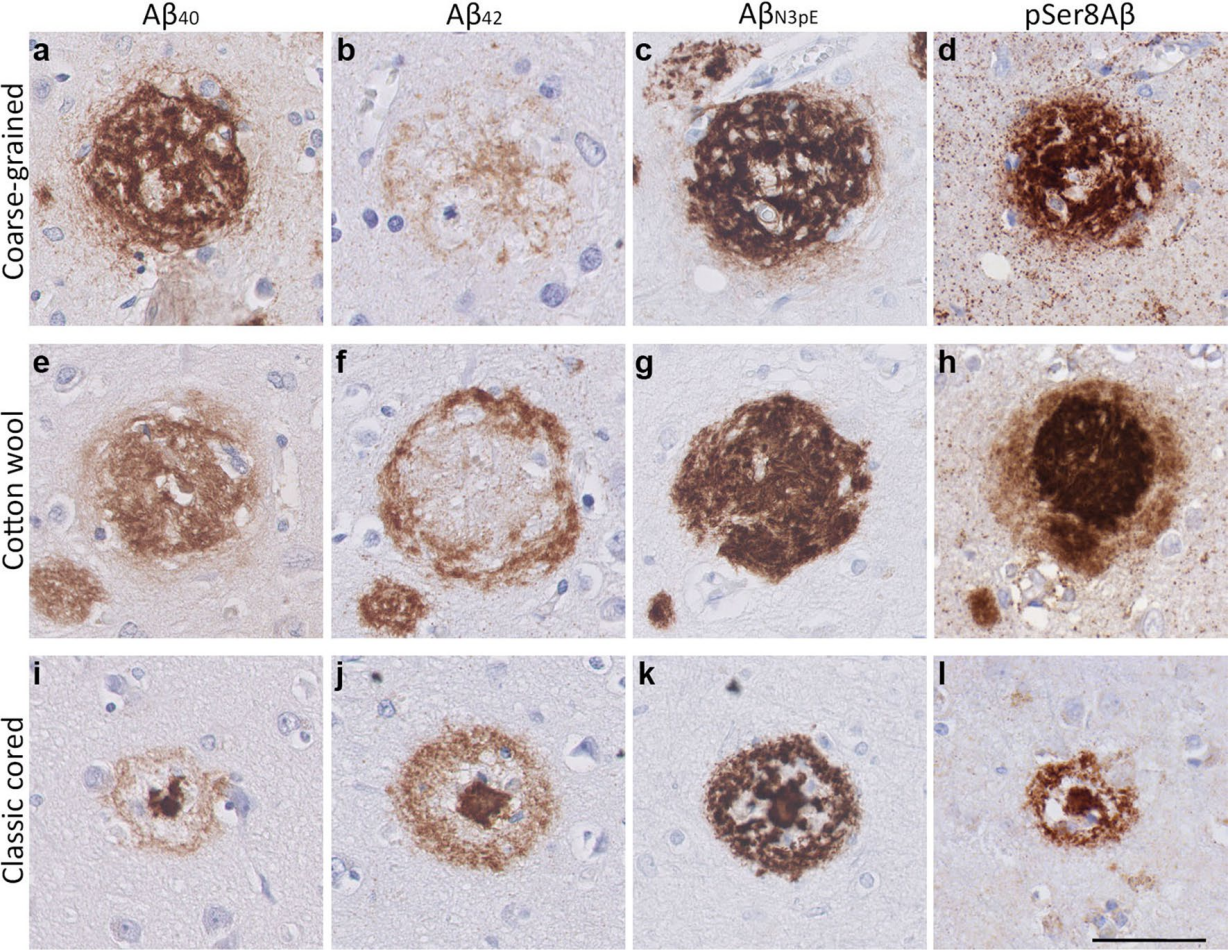
Aβ isoform composition of the coarse-grained plaque compared to the cotton wool and classic cored plaque. **a**, **b** The coarse-grained plaque was predominantly Aβ_40_ immunoreactive compared to Aβ_42_. **c**, **d** Strong immunostaining was also seen for Aβ_N3pE_ and pSer8Aβ. This Aβ composition differed from that of the cotton wool plaque (**e**–**h**) and the classic cored plaque (**i**–**l**). **e**, **f** The cotton wool plaque showed a central staining for Aβ_40_, that was surrounded by an Aβ_42_ immunoreactive ring. **g**, **h** The cotton wool plaque was immunoreactive for Aβ_N3pE_ and displayed a distinct halo immunoreactive for pSer8Aβ. **i**, **j** Classic cored plaques were predominantly Aβ_42_ compared to Aβ_40_ immunoreactive. **k**, **l** Both Aβ_N3pE_ and pSer8Aβ were detected in the entire classic cored plaque. Scale bar represents 50 µm and is applicable to all images. *Aβ*_*40*_ amyloid-beta 40, *Aβ*_*42*_ amyloid-beta 42, *Aβ*_*N3pE*_ truncated pyroglutamate Aβ, *pSer8Aβ* phosphorylated Aβ at serine 8

**Fig. 6:**
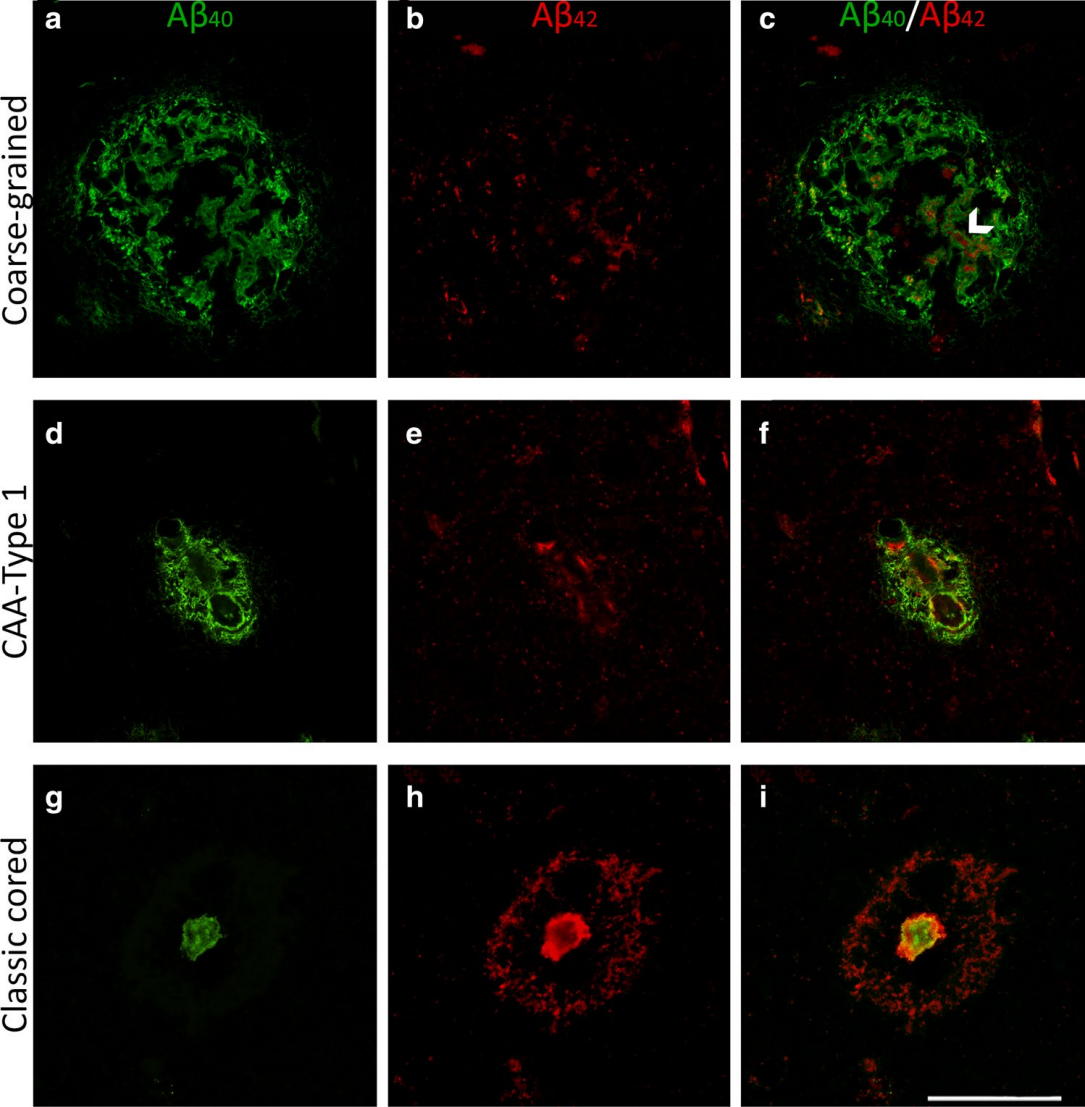
Immunofluorescence for Aβ_40_ and Aβ_42_ in the coarse-grained plaque compared to CAA-Type 1 and the classic cored plaque. **a**–**c** Double immunofluorescence labeling for Aβ_40_ (green) and Aβ_42_ (red) confirmed the Aβ_40_ predominance in coarse-grained plaques. **c** White arrowhead indicates an Aβ_40_ tubular-like structure filled with Aβ_42_. **d–f** CAA-Type 1 showed a similar Aβ_40_ to Aβ_42_ ratio as the coarse-grained plaque. **g–i** Classic cored plaques were predominantly Aβ_42_ immunoreactive. Scale bar represents 50 µm and is applicable to all images. *Aβ*_*40*_ amyloid-beta 40, *Aβ*_*42*_ amyloid-beta 42, *CAA* cerebral amyloid angiopathy

**Fig. 7:**
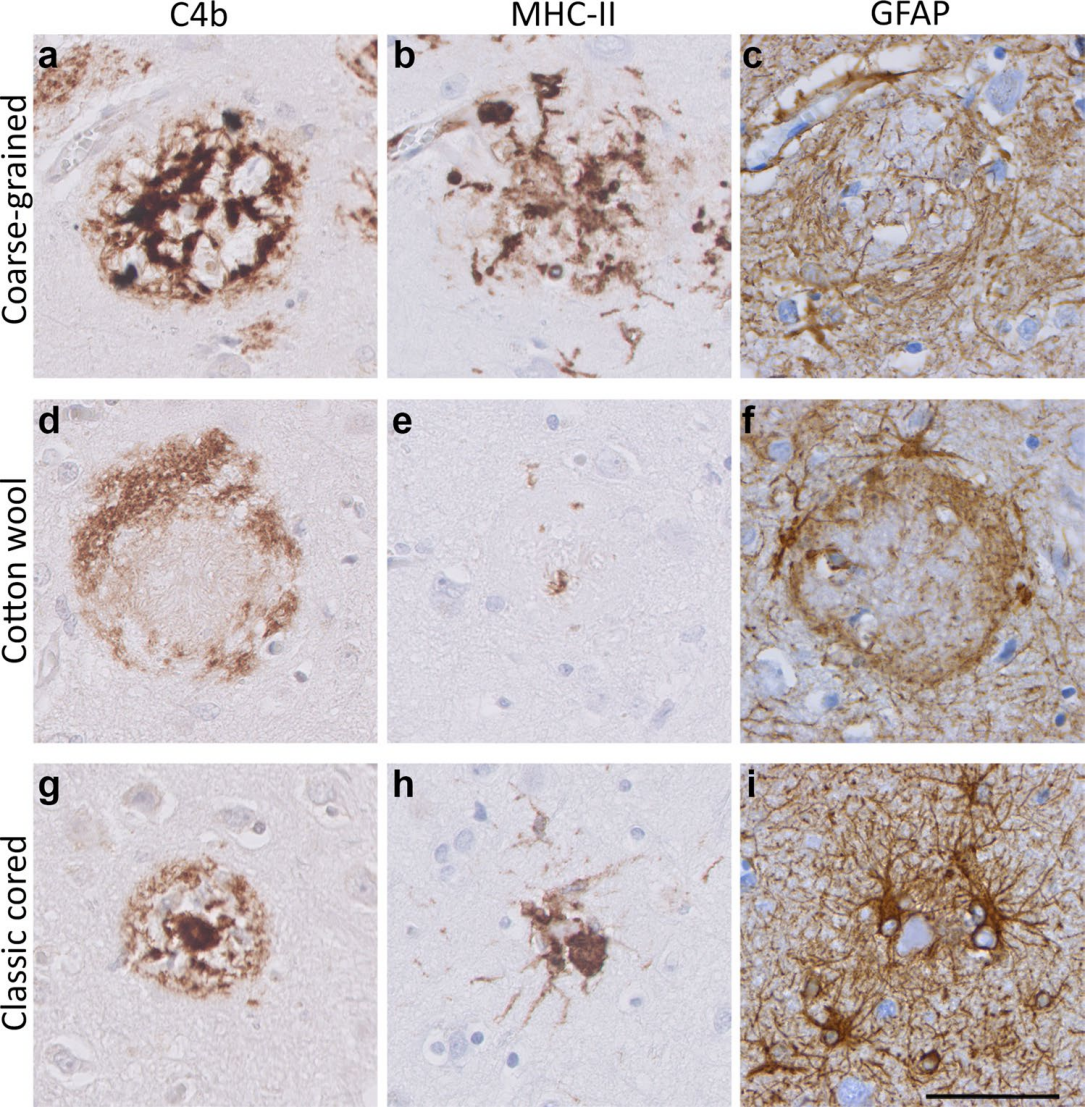
Neuroinflammatory response in the coarse-grained plaque compared to the cotton wool and classic cored plaque. **a**, **b** In the coarse-grained plaque, C4b was seen throughout the plaque with intense MHC-II immunoreactivity. **c** GFAP-positive astrocytes showed disrupted processes. **d**–**i** C4b, MHC-II and GFAP showed a different staining pattern in the cotton wool (**d**–**f**) and classic cored plaque (**g**–**i**). Scale bar represents 50 µm and is applicable to all images

**Fig. 8:**
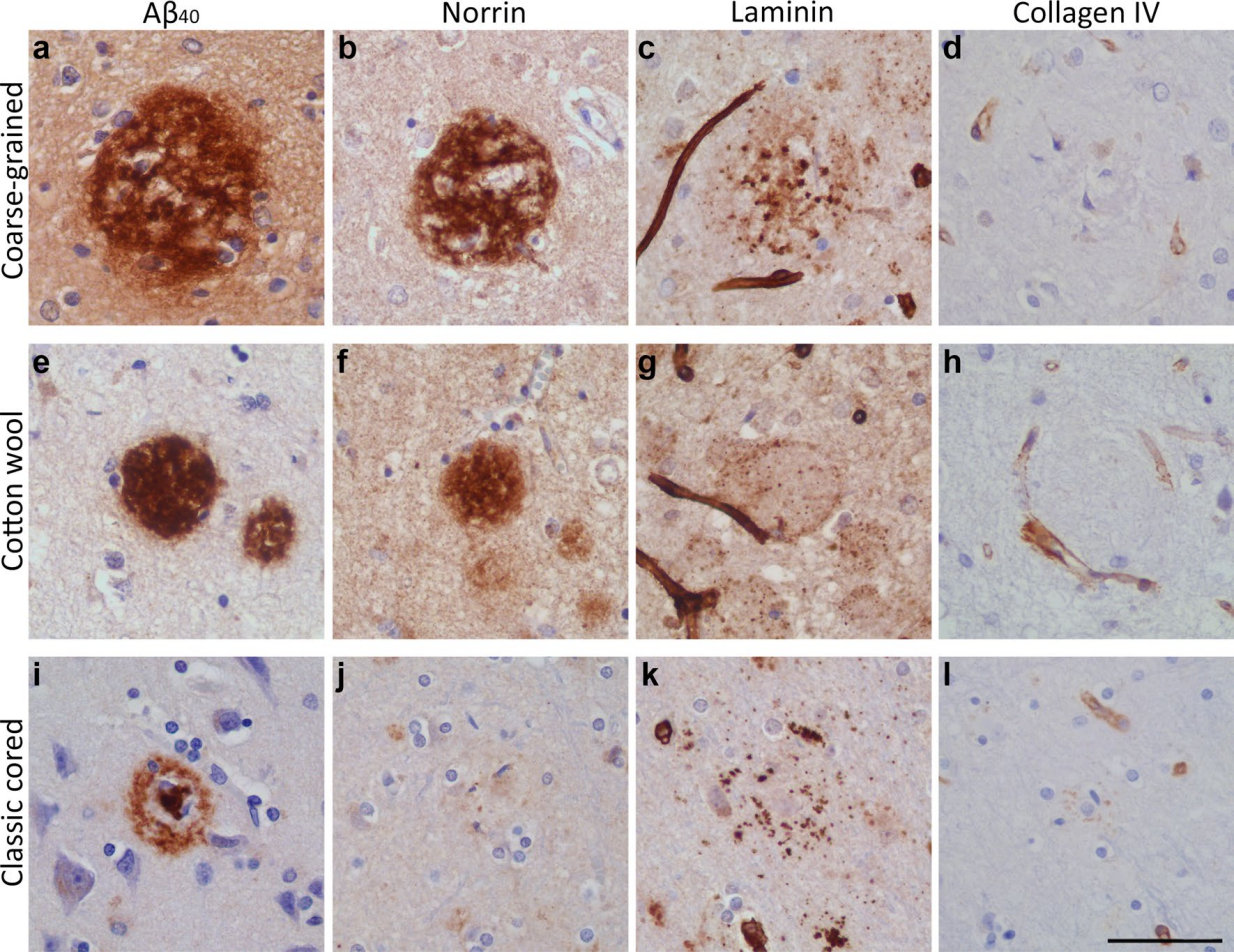
Vascular association of the coarse-grained plaque compared to the cotton wool and classic cored plaque. **a**–**d** Coarse-grained plaque showed immunoreactivity for Aβ_40_, norrin, and laminin but not for collagen IV. **e–h** Although the cotton wool plaque was positive for the same markers, staining morphology differed compared to the coarse-grained plaque. **i–l** Classic cored plaque has an Aβ_40_ positive core, was not immunoreactive for norrin, and showed a punctate staining for both laminin and collagen IV. Scale bar represents 50 µm and is applicable to all images. *Aβ*_*40*_ amyloid-beta 40

In-depth (immuno)histochemical characterization was performed by comparing the coarse-grained plaque to other clinically relevant plaques, i.e., the cotton wool plaque and the classic cored plaque (Figs. 3, 4, 5, 6, 7 and 8). See Table 2 for a complete summary of each (immuno)histochemical staining per investigated plaque-type. After H&E staining, the coarse-grained plaque showed tissue distortion without a sharply defined outline (Fig. 3b). The plaque contained amyloid fibrils as visualized by Congo red staining (Fig. 3c). Neuropil threads positive for pTau were always observed in and around the coarse-grained plaque (Fig. 3d). Dystrophic neurites positive for pTau were often, but not always visible within coarse-grained plaques. When present, the neurites were relatively less swollen compared to neurites seen in classic cored plaques. Also, the center of the coarse-grained plaque was devoid of pTau staining in the presence of dystrophic neurites. Of note, all cases with a high load of the coarse-grained plaque in the middle frontal gyrus section showed extensive pTau immunoreactivity. In addition, the coarse-grained plaque was, similarly to the classic cored plaque, immunoreactive for ApoE, APP, and PrP^C^ (Fig. 4). Immunohistochemical staining confirmed that the coarse-grained plaque was not immunoreactive for PrP^Sc^ (See Supplementary Material 1, Fig. S7, Online Resource).

The coarse-grained plaque has unique characteristics compared to the cotton wool and classic cored plaque (Fig. 3a–l). At first sight, the coarse-grained plaque looks similar to the cotton wool plaque due to its size and morphology observed with Aβ (aa 8-17; 6F/3D) immunostaining. However, the coarse-grained plaque had a less-defined border compared to the distinct circumscribed cotton wool plaque. The cotton wool plaque is visible in both Aβ (Fig. 3e) and in H&E as cotton wool-like patches (Fig. 3f). Furthermore, the cotton wool plaque is only faintly visible in Congo-red (Fig. 3g) and seldom contains dystrophic neurites (Fig. 3h). The coarse-grained plaque also differed from the classic cored plaque (Fig. 3i–l). The most obvious difference is in Ø and morphology, while the Ø of classic cored plaques is commonly 30–50 µm with a central core surrounded by a corona of diffuse Aβ, the Ø of the coarse-grained plaques is mostly 50–100 µm and lacks this central core. Both plaque types contained fibrillar amyloid and could contain dystrophic neurites. In the classic cored plaque the swollen dystrophic neurites surround the amyloid that is condensed into a central core. In addition, classic cored plaques are more often observed in the deeper cortical layers (V–VI) than in the more superficial layers [14, 45].

**Table 2:** Staining summary per plaque-type

Staining	The coarse-grained plaque	The cotton wool plaque	The classic cored plaque
Aβ (aa 8-17; 6F/3D)			
H&E	Tissue distortion	Circumscript defined patches	Visible core
Congo red	++; fibrillar amyloid throughout the plaque	−; not fibrillar	++; fibrillar amyloid condensed into a core
pTau	++; neuropil threads +−; dystrophic neurites	++; neuropil threads	+−; dystrophic neurites
APP	+; dystrophic neurites	+−	+; dystrophic neurites
ApoE	++; throughout the plaque	++; throughout the plaque	++; in the core; +−; in the corona
PrP^C^	+; throughout the plaque	+; throughout the plaque	++; in the core; +−; in the corona
Aβ_40_	++; throughout the plaque as fibrillary or tubular structures, 61% as shell surrounding the lesser Aβ_42_	++; homogenous throughout the plaque	++; in the core; +−; in the corona
Aβ_40_	+−; in the plaque center, sometimes co-localizing with Aβ_40_	+; outer ring	++; in both the core and the corona
Aβ_N3pE_	++	++	++
pSer8Aβ	++	++; intense stained plaque with diffuse halo	++
C4b	++; throughout the plaque	+; outer ring	+; in the core; +−; in the corona
CD68	++; within Aβ-devoid pores	+−; occasionally 1 cell body within the plaque	+; in-between the core and the corona
MHC-II	++; within Aβ-devoid pores	+−; occasionally 1 cell body within the plaque	+; in-between the core and the corona
GFAP	+; cell bodies are often found within the plaque	+; disrupted processes mostly staining the outer plaque edges	+
Norrin	++; fibril-like throughout the plaque	++; homogenous throughout the plaque	−
Laminin	+; small punctate dots throughout the plaque	+; small punctate dots throughout the plaque	+; small punctate dots surrounding the core
Collagen IV	−	−	+−; small punctate dots surrounding the core

(Immuno)histochemical staining summary per plaque-type is given and indicated as follows: −, no staining; +−, some positive staining; +, positive staining; ++, prominent positive staining. Supplementary Material 1, Table S1, Online Resource, for antibody and staining details

### The coarse-grained plaque has a distinct Aβ isoform composition, being predominantly Aβ_40_ immunoreactive

The coarse-grained plaque showed strong immunoreactivity for Aβ_40_, Aβ_N3pE_, and pSer8Aβ throughout the entire plaque (Fig. 5a, c, d). Interestingly, the coarse-grained plaque showed only little Aβ_42_ positivity (Fig. 5b). This weak Aβ_42_ immunoreactivity was observed more in the center than in the periphery of the plaque. Quantitative ELISA on laser-dissected coarse-grained plaques in two cases with a high load confirmed the relatively higher levels of Aβ_40_ compared to Aβ_42_. The coarse-grained plaques in case #18 contained 324.94 pg/µl of Aβ_40_ and 13.59 pg/µl of Aβ_42_, making the Aβ_40_/Aβ_42_ ratio = 23.91. The coarse-grained plaques in case #32 contained 264.24 pg/µl of Aβ_40_ and 9 pg/µl of Aβ_42_, making the Aβ_40_/Aβ_42_ ratio = 29.36.

The Aβ isoforms composition and distribution in the coarse-grained plaque were different from that of other plaques (Fig. 5e–h for cotton wool plaque and Fig. 5i–l for classic cored plaque). Similar to the coarse-grained plaque, the cotton wool plaque was predominantly Aβ_40_, Aβ_N3pE_, and pSer8Aβ positive. However, in the cotton wool plaque, the Aβ_42_ was observed as a small outer ring surrounding the Aβ_40_-positive center. In the cotton wool plaque, pSer8Aβ immunoreactivity showed a dense center surrounded by a less prominent halo. Aβ_N3pE_ distribution in cotton wool plaques was similarly as in coarse-grained plaques seen throughout the plaque. The difference between the coarse-grained and classic cored plaque was most prominent in their Aβ_40_ and Aβ_42_ composition. The classic cored plaque consisted predominantly of Aβ_42_, which was seen both in the core and corona of the plaque. Aβ_40_ was mostly seen in the core and occasionally little in the corona.

Although the location of coarse-grained plaques is different from that of CAA, we directly compared the Aβ_40_ and Aβ_42_ composition of the coarse-grained plaque to that of CAA due to a similar Aβ_40_ -predominance (Fig. 6). The classic cored plaque was used as a reference for plaque Aβ-isoform composition. In coarse-grained plaques, Aβ_40_ staining was somewhat tubular or trabecular-like. The Aβ_40_ immunoreactive trabeculae occasionally surrounded the more rare Aβ_42_ (Fig. 6c arrowhead). The coarse-grained plaque Aβ_40_ to Aβ_42_ ratio resembled that what is seen in CAA-Type 1, which showed a predominant presence of Aβ_40_ (Fig. 6d–f). As shown earlier, this was quite the opposite from what is seen in classic cored plaques (Fig. 6g–i).

### The coarse-grained plaque is associated with intense neuroinflammation and vascular pathology

To study Aβ-related disease mechanisms, immunostainings were performed for neuroinflammatory markers against complement (complement factor C4b), activated microglia (MHC-II/CD68), and reactive astrocytes (GFAP). These well-established markers have been shown to increase with AD pathology and are strongly associated with Aβ deposition [7, 24, 25]. The coarse-grained plaque showed intense immunoreactivity for C4b and activated microglia (Fig. 7a, b). C4b was seen throughout the plaque in a similar pattern as Aβ as observed in our previous study [7]. MHC-II staining showed intense microglial activation covering the complete coarse-grained plaque. GFAP staining indicated the presence of reactive astrocytes around the coarse-grained plaque with mostly disrupted GFAP-positive processes within the plaque (Fig. 7c).

The neuroinflammatory response appeared to be different in the coarse-grained plaque compared to the cotton wool (Fig. 7d–f) or classic cored plaque (Fig. 7g–i). In the cotton wool plaque, C4b (Fig. 7d) showed a ring-like immunostaining resembling the previous mentioned Aβ_42_ staining (Fig. 5f). Microglial activation was negligible and astrocytic processes seemed to encapsulate the cotton wool plaque (Fig. 7e, f, respectively). In the classic cored plaque, C4b was found predominantly in the core and to a lesser extent in the corona and activated microglia were located between the Aβ core and corona. Astrocytic processes in the classic cored plaque seemed less disrupted than in the coarse-grained plaque. In Supplementary Material 1, Fig. S8, Online Resource, comparable results for all 3 plaque types are shown but then visualized by triple immunofluorescence staining for C4b, CD68, and GFAP.

To study if the coarse-grained plaque was related to vascular pathology, we immunohistochemically stained sequential sections for Aβ_40_, the capillary-pathology-associated marker norrin [23], and vascular basement membrane markers (laminin and collagen IV [53]) (see Fig. 8). The coarse-grained plaque was both Aβ_40_ and norrin positive throughout the entire plaque. Anti-laminin staining showed small punctate dots in areas, where coarse-grained plaques were found in the adjacent section. Collagen IV was not observed in coarse-grained plaques. The same markers were assessed in the cotton wool plaque (Fig. 8e–h) and the classic cored plaque (Fig. 8i–l). The most striking difference was observed after immunostaining for norrin. Although cotton wool plaques also showed norrin immunoreactivity, the staining had a more homogenous appearance than in coarse-grained plaques. Classic cored plaques were not immunoreactive for norrin. All 3 plaque types showed a punctate staining for laminin (Fig. 8c, g, k). Whereas in coarse-grained plaques the laminin dots were seen throughout the plaque, in cotton wool plaques the dots were visible at the outer edges and in classic cored plaques the dots surrounded the core. Only in classic cored plaques, collagen IV positivity was sometimes vaguely seen as punctate dots surrounding the core (Fig. 8d, h, l). In Supplementary Material 1, Fig. S9, Online Resource, comparable results for all 3 plaque types are shown but then visualized by triple immunofluorescence staining for Aβ aa 1-16, norrin, and laminin.

### 3D visualization of the coarse-grained plaque

To gain a deeper understanding of the coarse-grained plaque, we scanned the plaque in 3D using high-resolution CLSM in the right middle frontal gyrus of 5 different cases with coarse-grained plaques (Supplementary Material 1, Table S1 for case details; Supplementary Material 1, Table S3 for # plaques per individual case, Online Resource). The combination of markers that was scanned was (1) Aβ_40_ & Aβ_42_; (2) Aβ, CD68, GFAP; (3) Aβ, norrin, laminin. Important to note for 3D interpretation is that due to fixation and pretreatment the tissue deforms and shrinks, especially in z-direction [15]. As a result, the originally 60 µm cut FFFF sections measured after mounting approximately 40 µm for combination 1 (Aβ_40_ & Aβ_42_) and 30 µm for combination 2 (Aβ, CD68, GFAP) and 3 (Aβ, norrin, laminin). Due to this ‘flattening’ in z-direction, the plaque, which is assumed to be a sphere, appeared as an ovoid.

#### 3D analysis of large coarse-grained plaques reveals an Aβ_40_ shell structure

**Fig. 9:**
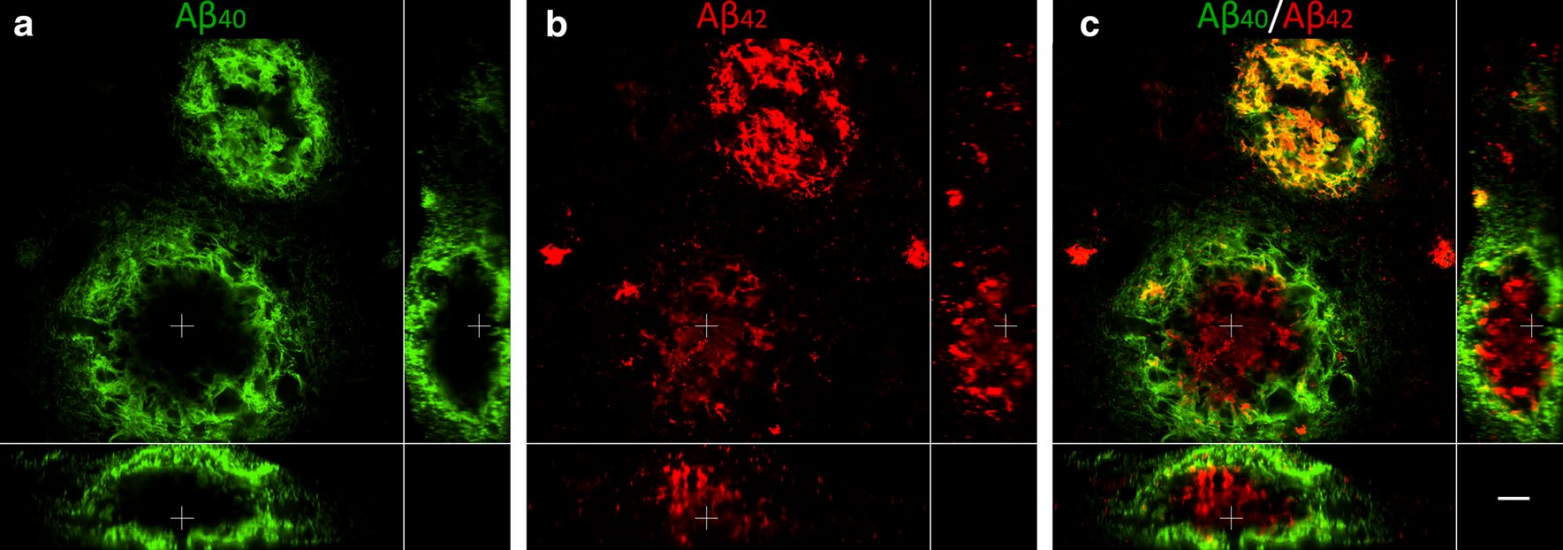
3D composition of Aβ_40_ and Aβ_42_ in the coarse-grained plaque. CLSM 3D image of two coarse-grained plaques stained for Aβ_40_ (**a**; green) and Aβ_42_ (**b**; red) with Aβ_40_/Aβ_42_ overlay in **c**, is shown. In the smaller coarse-grained plaque (upper plaque) Aβ_40_ and Aβ_42_ is co-localized. In the larger coarse-grained plaques (lower plaque) Aβ_40_ showed an outer shell structure, surrounding the lesser present Aβ_42_. White cross in XY indicates the same point in space as the white cross in XZ and YZ. Scale bar represents 10 µm and is applicable to all images. *Aβ*_*40*_ amyloid-beta 40, *Aβ*_*42*_ amyloid-beta 42, *CLSM* confocal laser scanning microscopy

A total of 38 coarse-grained plaques stained for Aβ_40_ & Aβ_42_ were scanned (see Supplementary Material 1, Table S3, Online Resource). 3D CLSM imaging showed that the predominant Aβ_40_ staining showed a trabecular-like and sometimes even tubular-like morphology in the coarse-grained plaque, confirming our 2D observations (Fig. 9 and Supplementary Materials 2 and 3, Online Resource). This trabecular appearance was most prominent at the surface of the plaque. Interestingly, in 61% of all scanned coarse-grained plaques, the Aβ_40_ formed an outer shell around the lesser Aβ_42_ (Supplementary Material 1, Table S3, Online Resource). This was especially prominent in the larger coarse-grained plaques (Ø ≈ 80 μm; larger plaque in Fig. 9 and Supplementary Material 2, Online Resource, and the plaque in Supplementary Material 3, Online Resource). In the smaller coarse-grained plaques (Ø ≈ 50 μm), the Aβ isoform segregation was less present and Aβ_40_ and Aβ_42_ more often co-localized (smaller plaque in Fig. 9, and Supplementary Material 2, Online Resource).

#### Neuroinflammatory response is seen throughout the coarse-grained plaque

**Fig. 10:**
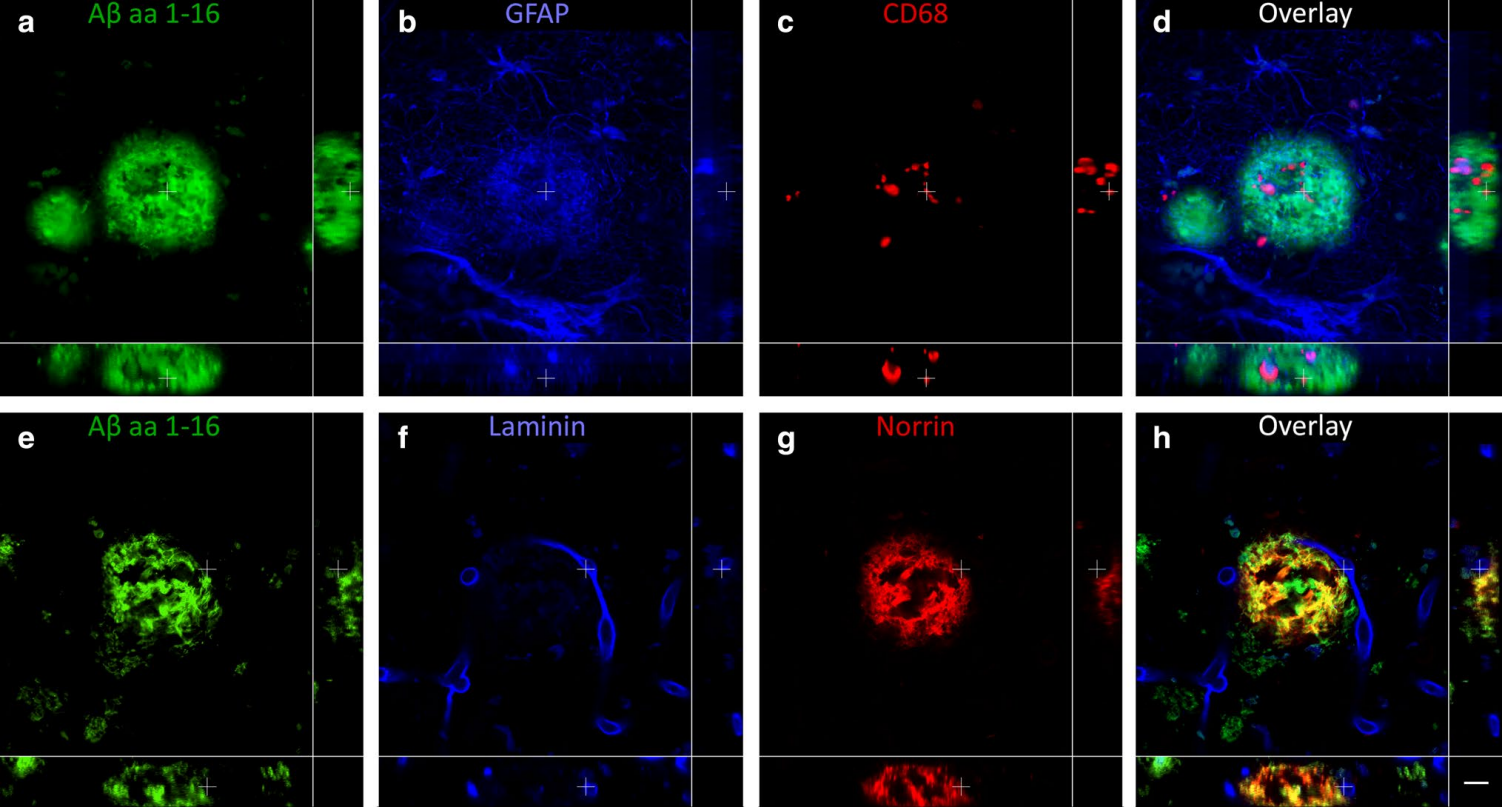
3D composition of neuroinflammation and vascular attribution in the coarse-grained plaque. Representative CLSM 3D images are shown for neuroinflammation (top row) and vascular attribution (bottom row) in and near the coarse-grained plaque. CD68 or GFAP immunoreactivity was found throughout the coarse-grained plaque (top row). Most coarse-grained plaques were in direct contact with a vessel, which appeared as a string vessel for this particular plaque. White cross in XY indicates the same point in space as the white cross in XZ and YZ. Scale bar represents 10 µm and is applicable to all images. *Aβ* amyloid-beta, *CLSM* confocal laser scanning microscopy

A total of 36 coarse-grained plaques stained for neuroinflammatory markers (Aβ, CD68, and GFAP) were scanned (Supplementary Material 1, Table S3, Online Resource). Although not all Aβ-devoid pores were filled with CD68 or GFAP, both markers were found throughout the plaque in a rather unorganized manner, confirming our 2D-based observations (Fig. 10 upper row a–d; Supplementary Materials 4 and 5, Online Resource).

#### 3D analysis hints to a vascular component in the coarse-grained plaque

Forty-four coarse-grained plaques stained for vascular markers (Aβ, norrin, and laminin) were scanned (Supplementary Material 1, Table S3, Online Resource). Based on 2D observations of the plaque’s Aβ_40_ and norrin immunoreactivity, the tubular structures, and the plaque’s association with CAA-affected vessels, we expected CAA vessels to penetrate the coarse-grained plaque in 3D. This was, however, not observed with CLSM analysis. We did observe that at least 37/44 (84%) of scanned coarse-grained plaques were in direct contact with a vessel (Fig. 10 bottom row e-h; Supplementary Materials 6 and 7, Online Resource). Seven of the 44 scanned plaques were not in direct contact with a vessel. Five of those 7 could not be completely scanned in z-direction, and therefore, their potential vessel connection could be missed, making the 84% most likely an underestimation. A little bulge in laminin, possibly reflecting the localization of a pericyte (mean width **≈** 7 μm) [4], was often observed, where the plaque touched the vessel. Although the touching vessel was not immunoreactive for norrin, norrin positive threads within the plaque were closely connected to the vessel (Supplementary Materials 6 and 7, Online Resource). In addition, similar to what was observed in 2D DAB staining, laminin staining was seen as punctate dots although with lesser intensity.

## Discussion

In this study we defined a divergent plaque-type, called the coarse-grained plaque, with distinct characteristics compared to earlier described plaques. Using anti-Aβ immunostaining, the coarse-grained plaque was defined by its size (30–100 µm), multi-cored coarse-grainy appearance, Aβ-devoid pores, and an ill-defined border. Increased presence of the coarse-grained plaque was related to an early disease onset in AD, a homozygous *APOE* ε4 status, and the presence of CAA-Type 1. The coarse-grained plaque was not observed in Aβ-positive clinically non-demented cases. Together, these results highlight the association of this plaque-type with the clinical manifestation of AD. In-depth characterization revealed that the plaque contains fibrillar amyloid, was mostly composed of Aβ_40_, and showed strong immunoreactivity for neuroinflammation- as well as vascular pathology-associated markers. In-depth 3D assessment exposed an Aβ_40_ shell structure in the larger coarse-grained plaques and a direct vascular connection.

**Fig. 11:**
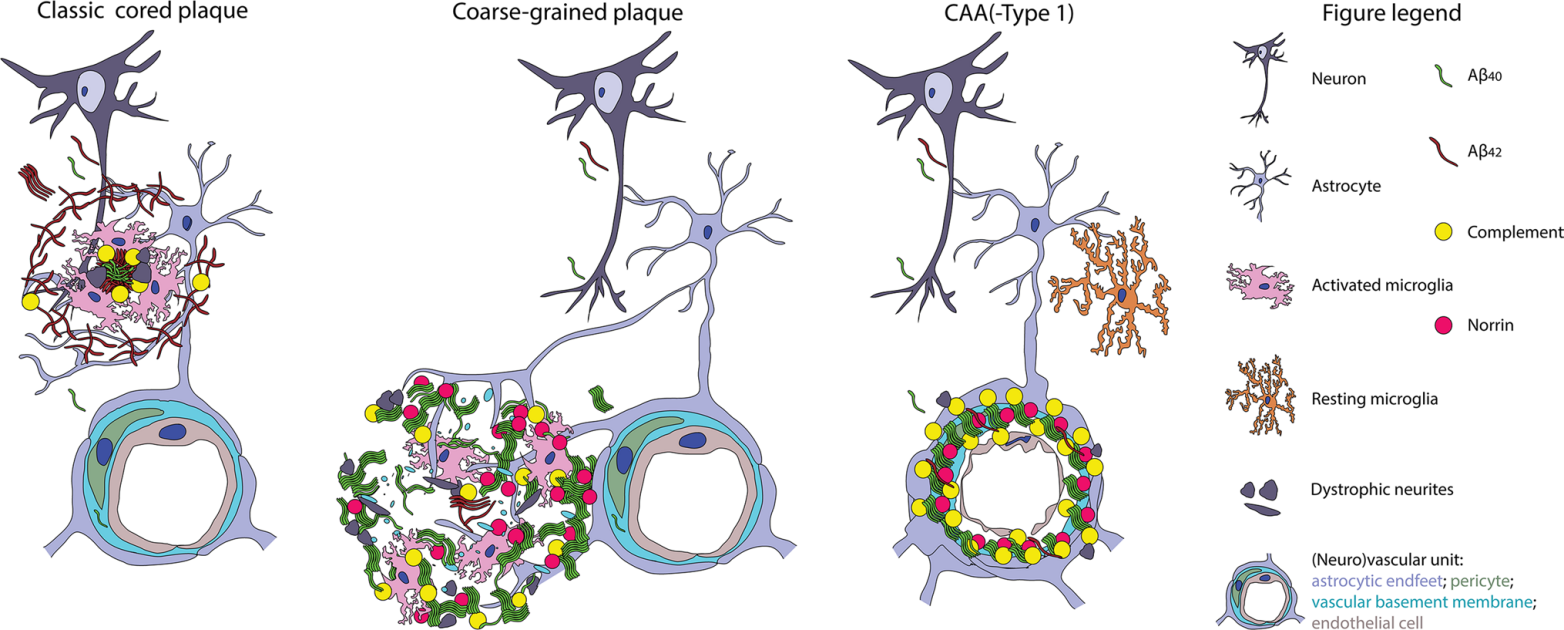
Hypothetical illustration of the coarse-grained plaque’s origin. This figure illustrates the coarse-grained plaques’ similarities and differences with the two other forms of Aβ deposits, being parenchymal plaques illustrated by the classic cored plaque and vascular-located Aβ illustrated by CAA. We placed the coarse-grained plaque in-between parenchymal and vascular aggregates, based on its parenchymal perivascular localization, microglial activation, Aβ_40_ predominance and norrin immunoreactivity. See “Discussion” section for explanation. *Aβ* amyloid-beta, *Aβ*_*40*_ amyloid-beta 40, *Aβ*_*42*_ amyloid-beta 42, *CAA* cerebral amyloid angiopathy

Based on the plaque’s characteristics observed in this study, we hypothesize that the coarse-grained plaque evolves at the parenchymal border of the capillary blood–brain barrier as illustrated in Fig. 11. To discuss the placement of the coarse-grained plaque, we made a direct comparison with the classic cored plaque and CAA-Type 1 as a reference for parenchymal and vascular deposited Aβ, respectively. In the classic cored plaques predominant Aβ_42_ precipitates, after neuronal excretion, in the brain parenchyma, most likely due to the increased aggregation properties and decreased drainage of Aβ_42_ compared to Aβ_40_ [28]. The aggregated Aβ becomes increasingly fibrillar and is then associated with complement factors, reactive astrocytes, and activated microglia [7, 17, 26, 54]. Dystrophic neurites immunoreactive for APP or pTau can be observed near the amyloid containing aggregates [16]. In case of CAA, the excessive Aβ is also produced neuronally [22]. However, the excreted Aβ than consists mainly of Aβ_40_, which is hypothesized to travel further than the plaque-associated Aβ_42_ due to increased solubility and only aggregates once it reaches the (peri)vasculair drainage system [22]. The complement cascade becomes also activated and dystrophic neurites can be found surrounding the congophilic vessels [37, 38, 56]. However, different from plaques, microglial and macrophage activity markers in CAA do not seem to be increased compared to control vessels [56]. Only when Aβ is found to be dyshoric, meaning the Aβ is deposited in the parenchyma around the amyloid-laden vessel, microglia activation is present in CAA [41]. When CAA is located in the capillaries, referred to as CAA-Type 1, the vessels are immunoreactive for the norrin protein [23]. We placed the coarse-grained plaque between the classic cored plaque and CAA-Type 1, since it showed similarities and differences with both types of Aβ deposits. Similar to the classic cored plaque, the coarse-grained plaque is localized within the brain parenchyma and is immunoreactive for complement and associated with activated microglia and astrocytes [7]. The amyloid structure of the coarse-grained plaque most likely favours a strong binding and activation of complement factors, which in turn could act as opsonins for phagocytosis carried out by microglia [33, 46, 57]. APP and PrP^C^ immunoreactivity in the coarse-grained plaque could reflect dystrophic neurites, indicating damage to the surrounding axons. Similar to CAA-Type 1, the coarse-grained plaque mainly consists of Aβ_40_ and is immunoreactive for norrin [6, 23]. Different to CAA, the vascular morphology in coarse-grained plaques is not so obvious. However, using anti-Aβ immunostaining we did observe tubular-like structures in the coarse-grained plaque. Together with the dot-like laminin immunoreactivity, we speculate these tubular structures to be vascular remnants with the laminin representing the collapsed vascular basement membrane. Furthermore, the coarse-grained plaque was predominantly observed in sulcal fundi, in which the vascular density is higher than in gyral crowns [2]. Although CAA-affected vessels were often noted in the proximity of coarse-grained plaques and most coarse-grained plaques were in direct contact with a vessel, these vessels were not affected by CAA nor did they penetrate the plaque. For these reasons, we assume that the Aβ aggregation in coarse-grained plaque does not start within the vessel wall as in CAA, but rather starts at the parenchymal border of the capillary blood–brain barrier, which is similar to the location of dyshoric Aβ in CAA-Type 1. Why the Aβ_40_ in case of CAA is capable of crossing the blood–brain barrier and in case of the coarse-grained plaque is not, remains elusive. The observed Aβ_40_ shell structure surrounding the lesser Aβ_42_ in larger coarse-grained plaques is remarkable. Although we are unsure of the essence of the shell structure, we hypothesize it to be related to the aggregation properties and the development of the coarse-grained plaque.

Similar amyloid plaque structures as what we define here as the coarse-grained plaque have sporadically been described by others. The coarse-grained plaque is comparable to the previously described ‘fibrous’ or ‘fibrillar’ plaque described by Schmidt et al. and Dickson & Vickers, respectively [14, 45]. Although studied in a small cohort, both groups show that this type of plaque is associated with clinical symptoms of dementia, which is in line with our results. This association was not found for the classic cored plaque, since this plaque was also observed in cases without clinical symptoms [14]. Furthermore, Schmidt et al. concluded that these fibrous—or what we deemed ‘coarse-grained’—plaques are rare in AD, but are particular common in Down syndrome cases with AD pathology [45]. With a mean age of death at 78 years, it is most likely that the AD cohort studied by Schmidt et al. mainly consisted of LOAD cases. After the reported observation 25 years ago, the coarse-grained plaque escaped attention and was not included in the currently most used plaque categorization scheme [48, 51]. To our knowledge, we are the first to perform such in-depth characterization for the coarse-grained plaque and to report that this plaque is especially prominent in EOAD.

As Aβ pathology starts long before clinical symptoms arise and comes in a myriad of different morphologies [27, 48], it is important to study Aβ deposits that are clinically relevant. The fact that coarse-grained plaques were only observed in cases with clinical symptoms of AD and not in non-demented Aβ-positive cases, indicates the clinical relevance of this plaque type. In addition, the coarse-grained plaque contains Aβ isoforms that correlate with clinical disease progression. Previously, Rijal Upadhaya et al. showed that both Aβ_42_ and Aβ_40_ were detected in brain homogenates of Aβ-positive but cognitively-healthy cases [43]. In cases with pre-clinical AD, Aβ_N3pE_ was also detectable, while pSer8Aβ was only detected in cases with clinical dementia due to AD. The immunoreactivity of the coarse-grained plaque for pSer8Aβ is in line with the observation that this plaque is only seen in clinically demented cases. This could be a relevant finding for exploring biomarkers reflecting specific Aβ deposits that could distinguish between clinical subgroups in AD .

As EOAD cases are likely to be genetically predisposed, the occurrence of the coarse-grained plaque could be related to genetic factors. However, extensive genetic screening for the known mendelian mutations in *APP*, *PSEN1*, and *PSEN2* for AD could not link the coarse-grained plaque to the functional impairment in one of those genes such as found for the cotton wool plaque, which is linked to an exon 9 deletion in the *PSEN1* gene [10]. We did observe an association with the most prevalent genetic risk factor for AD, being *APOE* ε4. Interestingly, mice transgenic for human *APOE* ε4 and a familial *APP* mutation (E4FAD mice) showed both larger plaques and increased neuroinflammation compared to *APOE* ε4 negative FAD mice [2]. Although at first sight the plaques stained with thioflavin S in E4FAD mice may resemble the coarse-grained plaque, the mouse plaques were predominantly Aβ_42_ positive, which is an important difference with the coarse-grained plaque. In our view it is too soon to draw any conclusions on the functional correlation with *APOE* ε4, especially since at least one-third of the cases with a frequent degree of coarse-grained plaques lacked any ε4 allele. This makes it most likely that besides *APOE* also other (genetic) factors come into play. Since the coarse-grained plaque was more prevalent in EOAD than in LOAD, the plaque is likely to be genetically predisposed. Therefore, it would be interesting for future studies to investigate whether we can identify additional genetic mutations or variants in individuals with coarse-grained plaques.

Some limitations may apply to this work. We are well aware that post-mortem studies are cross-sectional, making our hypothesis on plaque-development speculative. It would be interesting to study the plaque’s development in a mechanistic model. However, this would require more in-depth background on the pathology (e.g., proteome) of the plaque, the genotype, and the clinical phenotype of cases with coarse-grained plaques. In this study, CD68 was used as a microglial activation marker in 3D visualization. It would be interesting to extend this microglia marker panel with other activation markers as well as more microglia specific markers (e.g., P2Y12, TMEM119, Iba1) to gain better insights on the microglial involvement.

We observed the coarse-grained plaque to be especially prominent in the neocortex, which is one of the first regions to develop Aβ pathology [51]. The coarse-grained plaque, similar to other plaques, is likely to be region specific [7, 51]. In this study we observed that the coarse-grained plaque was predominantly found in the frontal and parietal regions and to a lesser extend also in the temporal and occipital regions of the neocortex. Future work could focus on the presence of coarse-grained plaques in different pathology stages, as well as in different clinical AD phenotypes.

## Conclusion

In this study, we describe and define a new type of Aβ deposit, referred to as the ‘coarse-grained plaque’. Its characteristics are different from other Aβ deposits. We provide a morphological and biochemical definition for the coarse-grained plaque, supporting that this deposit is unique, with specific clinical and etiological associations. Associated disease mechanisms such as neuroinflammation and vascular attribution, as well as the structure and biochemical composition of Aβ may lie at the cause of morphological differences between Aβ deposits. Disentangling specific Aβ deposits between AD subgroups may be important in the search for disease-mechanistic-based therapies in the near future.

## Electronic supplementary material

Below is the link to the electronic supplementary material.

Supplementary material 1 (DOCX 21834 kb)

Supplementary material 2 (MP4 158391 kb)

Supplementary material 3 (MP4 139258 kb)

Supplementary material 4 (MP4 221514 kb)

Supplementary material 5 (MP4 136527 kb)

Supplementary material 6 (MP4 180933 kb)

Supplementary material 7 (MP4 134703 kb)
