# Supplementary material for: The coarse-grained plaque: a divergent Aβ plaque-type in early-onset Alzheimer’s disease
Source: Acta Neuropathol. 2020 Sep 14;140(6):811–30. doi: 10.1007/s00401-020-02198-8 (PMC7666300; doi:10.1007/s00401-020-02198-8)
Supplement: Supplementary file 1 — Supplementary material 1 (DOCX 21834 kb) [file 401_2020_2198_MOESM1_ESM.docx]

**Supplementary Table S1** Case details

| Case # | Group | Gender | Onset age | Disease duration | Age † | Cause † | PMI | *APOE* | Pathogenic mutation in *APP*, *PSEN1*, or *PSEN2* | ABC [35] | Thal Aβ phase [51] | Braak NFT stage [8] | CAA-Type [49] | Braak LB stage [1] | LATE-NC [36] | Vascular lesions | Coarse-grained plaque score | FFPE region | FFFF region |
| --- | --- | --- | --- | --- | --- | --- | --- | --- | --- | --- | --- | --- | --- | --- | --- | --- | --- | --- | --- |
| 1 | Aβ + | Male | NA | NA | 82 | Lung carcinoma | 05:30 | NA | NA | A1B1C0 | 1 | 2 | 1 | NA | NA | None | None | MFG |  |
| 2 | Aβ + | Male | NA | NA | 93 | Heart failure | 08:30 | NA | NA | A1B2C0 | 1 | 3 | 2 | 6 | 1 | None | None | MFG |  |
| 3 | Aβ + | Male | NA | NA | 82 | Euthanasia | 07:30 | 34 | NA | A1B1C0 | 1 | 1 | 0 | 4 | 0 | None | None | MFG |  |
| 4 | Aβ + | Female | NA | NA | 64 | Relapsing pneumothorax | 05:40 | 23 | NA | A1B0C0 | 1 | 0 | 0 | 0 | NA | None | None | MFG |  |
| 5 | Aβ + | Female | NA | NA | 91 | Brain stem infarction | 03:47 | 33 | NA | A1B1C0 | 2 | 2 | 2 | 0 | NA | Multiple i’s | None | MFG |  |
| 6 | Aβ + | Female | NA | NA | 89 | Euthanasia | 06:30 | 34 | NA | A1B1C0 | 1 | 3 | 2 | 0 | NA | Multiple i’s | None | MFG |  |
| 7 | Aβ + | Male | NA | NA | 71 | Pancreas carcinoma | 08:55 | 34 | None | A2B1C1 | 3 | 2 | 2 | 0; Amygdala only | NA | None | Sparse | MFG |  |
| 8 | Aβ + | Female | NA | NA | 70 | Pneumonia by pulmonary carcinoma | 06:15 | 33 | None | A1B1C0 | 1 | 2 | 1 | 0 | NA | None | None | MFG |  |
| 9 | Aβ + | Female | NA | NA | 70 | Cachexia by pancreas carcinoma | 07:35 | 33 | None | A1B1C0 | 1 | 2 | 2 | 0 | NA | None | None | MFG |  |
| 10 | Aβ + | Female | NA | NA | 78 | Pneumonia | 04:35 | 33 | None | A1B2C1 | 2 | 3 | 2 | 0 | NA | Hypertensive vasculopathy; mi | None | MFG |  |
| 11 | Aβ + | Male | NA | NA | 79 | Euthanasia | 05:45 | 33 | None | A1B1C1 | 1 | 3 | 1 | 0 | NA | Calcified vessels in WM | None | MFG |  |
| 12 | Aβ + | Male | NA | NA | 79 | Euthanasia | 06:30 | 23 | None | A1B1C0 | 1 | 2 | 0 | 0; Amygdala only | NA | None | None | MFG |  |
| 13 | Aβ + | Male | NA | NA | 83 | Myocard infarct | 05:15 | 33 | None | A1B1C0 | 1 | 1 | 0 | 1 | NA | i | None | MFG |  |
| 14 | Aβ + | Male | NA | NA | 95 | Heart failure | 07:15 | 24 | None | A2B1C0 | 3 | 2 | 1 | 1 | NA | Hypertensive vasculopathy; multiple mi’s | None | MFG |  |
| 15 | Aβ + | Female | NA | NA | 78 | Euthanasia | 07:10 | 33 | None | A1B1C0 | 1 | 1 | 0 | 0 | NA | None | None | MFG |  |
| 16 | EOAD | Male | 56 | 8 | 64 | Cachexia/dehydration | 4:40 | 24 | None | A3B3C3 | ≥4 | 5 | 2 | 0 | NA | None | Sparse | MFG |  |
| 17 | EOAD | Female | 64 | 3 | 67 | Epileptic insult | 4:45 | 33 | None | A3B3C3 | ≥4 | 5 | 2 | 0 | NA | Gliotic scars | Sparse | MFG |  |
| 18 | EOAD | Male | 48 | 11 | 59 | Cachexia/dehydration | 7:35 | 44 | None | A3B3C3 | ≥4 | 6 | 1 | 0 | NA | None | Frequent | MFG |  |
| 19 | EOAD | Male | 59 | 6 | 65 | Pneumonia | 03:32 | 33 | NM_000021.3(PSEN1):c.791C>T (p.Pro264Leu) | A3B3C3 | 5 | 6 | 1 | 0; Amygdala only | NA | None | Sparse | MFG |  |
| 20 | EOAD | Male | 56 | 6 | 62 | Cachexia/dehydration | 04:40 | 33 | None | A3B3C3 | ≥4 | 5 | 1 | 0; Amygdala only | 0 | Small vessel disease | Sparse | MFG |  |
| 21 | EOAD | Male | 58 | 2 | 60 | Euthanasia | 08:35 | 33 | NM_000021.3(PSEN1):c.786G>C p.(Leu262Phe) | A3B3C3 | 5 | 6 | 1 | 0 | NA | None | Sparse | MFG |  |
| 22 | EOAD | Male | 62 | 6 | 68 | Euthanasia | 09:15 | 33 | None | A3B3C3 | 5 | 5 | 2 | 0 | NA | None | Sparse | MFG |  |
| 23 | EOAD | Female | 56 | 10 | 66 | Sedatives and pneumonia | 08:01 | 33 | None | A3B3C3 | 5 | 6 | 2 | 0 | 0 | Hypertensive vasculopathy; mi | Sparse | MFG |  |
| 24 | EOAD | Male | 58 | 7 | 65 | Cardiac arrest | 07:51 | 33 | NA | A3B3C3 | 4 | 5 | 1 | 0 | ≥2 | Multiple mi’s | Sparse | MFG |  |
| 25 | EOAD | Male | 64 | 9 | 73 | NA | 06:37 | 34 | NA | A3B3C3 | 5 | 6 | 1 | 0 | 0 | None | Sparse | MFG |  |
| 26 | EOAD | Female | 55 | 10 | 65 | Euthanasia | 06:41 | 33 | None | A3B3C2 | 5 | 5 | 2 | 0 | NA | None | None | MFG |  |
| 27 | EOAD | Male | 56 | 6 | 62 | Malign neuroleptic syndrome | 4:15 | 34 | None | A3B3C3 | ≥4 | 6 | 1 | 0 | NA | None | Moderate | MFG |  |
| 28 | EOAD | Male | 63 | 7 | 70 | Metastasized colon carcinoma | 6:20 | 34 | None | A3B3C3 | ≥4 | 6 | 1 | 0 | NA | None | Moderate | MFG; + |  |
| 29 | EOAD | Male | 54 | 8 | 62 | Palliative sedation | 08:15 | 34 | None | A3B3C3 | 5 | 6 | 1 | 0 | 0 | None | Moderate | MFG; + | MFG |
| 30 | EOAD | Male | 58 | 9 | 67 | Dehydration | 06:35 | 34 | None | A3B3C3 | 5 | 6 | 1 | 0 | NA | None | Moderate | MFG; + | MFG |
| 31 | EOAD | Female | 59 | 6 | 65 | Cachexia/dehydration | 5:40 | 33 | None | A3B3C3 | ≥4 | 6 | 2 | 0 | NA | None | Frequent | MFG |  |
| 32 | EOAD | Male | 60 | 5 | 65 | Cardiac insufficiency | 8:50 | 44 | None | A3B3C3 | ≥4 | 6 | 1 | 0 | NA | None | Frequent | MFG |  |
| 33 | EOAD | Male | 62 | 3 | 65 | Euthanasia | 6:50 | 34 | None | A3B3C3 | ≥4 | 5 | 1 | 0 | NA | None | Frequent | MFG; + |  |
| 34 | EOAD | Male | 52 | 9 | 61 | Pneumonia | 05:00 | 34 | None | A3B3C3 | 4 | 6 | 1 | 0; Amygdala predominant | NA | None | Frequent | MFG; + |  |
| 35 | EOAD | Male | 58 | 17 | 75 | Cachexia/dehydration | 03:05 | 44 | None | A3B3C3 | 5 | 6 | 2 | 0 | NA | None | Frequent | MFG; + |  |
| 36 | EOAD | Male | 53 | 11 | 64 | Pneumonia | 04:35 | 33 | None | A3B3C3 | 5 | 6 | 1 | 0; Amygdala predominant | NA | None | Frequent | MFG; + |  |
| 37 | EOAD | Male | 58 | 11 | 69 | Pneumonia | 11:55 | 34 | None | A3B3C3 | 5 | 5 | 1 | 0 | NA | None | Frequent | MFG; + |  |
| 38 | EOAD | Male | Before 58 | NA | 59 | Euthanasia | 06:31 | 44 | None | A3B3C3 | 5 | 5 | 1 | 5 | 0 | Extensive calcifications in basal nulcei and hippocampus | Frequent | MFG; + |  |
| 39 | EOAD | Female | 61 | 13 | 74 | Cachexia/dehydration | 05:15 | 44 | None | A3B3C3 | 5 | 6 | 2 | 0; Amygdala only | 0 | None | Frequent | MFG; + |  |
| 40 | EOAD | Female | 47 | 15 | 62 | Palliative sedation | 06:25 | 44 | NA | A3B3C3 | 5 | 6 | 1 | 0 | 0 | None | Frequent | MFG |  |
| 41 | EOAD | Male | 32 | 5 | 37 | Euthanasia | 11:11 | 23 | NM_000021.3(PSEN1):c.1254G>T (p.Leu418Phe) | A3B3C3 | 5 | 6 | 1 | 0; Amygdala only | 0 | None | Frequent | MFG; + |  |
| 42 | EOAD | Male | 53 | 5 | 58 | Cachexia/dehydration | 08:55 | 34 | None | A3B3C3 | 5 | 4 | 1 | 0 | 0 | None | Frequent | MFG; + | MFG |
| 43 | EOAD | Female | 56 | 15 | 71 | Pneumonia | 03:30 | 24 | None | A3B3C3 | ≥4 | 6 | 2 | 0 | NA | None | Sparse | MFG |  |
| 44 | EOAD | Male | 60 | 11 | 71 | Cachexia/dehydration | 06:35 | 33 | None | A3B3C3 | ≥4 | 6 | 1 | 0 | NA | None | None | MFG |  |
| 45 | EOAD | Female | 40 | 27 | 67 | Cerebrovascular accident | 04:30 | 34 | None | A3B3C3 | 4 | 6 | 2 | 4 | NA | i | Sparse | MFG |  |
| 46 | EOAD | Female | 54 | 7 | 61 | Cachexia/dehydration | 05:10 | 44 | None | A3B3C2 | ≥4 | 6 | 2 | 0 | NA | None | Moderate | MFG |  |
| 47 | EOAD | Female | 34 | 37 | 43 | Cachexia/dehydration | 04:15 | 33 | NM_000021.3(PSEN1):c.617G>A (p.Gly206Asp) | A3B3C3 | 5 | 6 | 2 | 0; Amygdala predominant | NA | None | Moderate | MFG |  |
| 48 | EOAD | Female | 60 | 13 | 73 | NA | 07:17 | 33 | None | A3B3C3 | 5 | 6 | 1 | 0; Amygdala only | NA | None | Frequent | MFG |  |
| 49 | EOAD | Male | 61 | 11 | 72 | Pneumonia and stomach bleed | 05:15 | 34 | None | A3B3C2 | 5 | 6 | 1 | 0; Amygdala predominant | NA | Lobar bl; i | Frequent | MFG |  |
| 50 | EOAD | Female | 64 | 27 | 91 | NA | 04:20 | 33 | None | A3B3C3 | 5 | 6 | 1 | 0 | NA | i’s | Frequent | MFG |  |
| 51 | EOAD | Male | 47 | 12 | 59 | Euthanasia | 05:25 | 33 | APP duplication | A3B3C3 | 5 | 5 | 1 | 0; Amygdala only | NA | None | Frequent | MFG |  |
| 52 | EOAD | Female | 51 | 21 | 70 | Cachexia/dehydration | 04:20 | 44 | None | A3B3C3 | 5 | 6 | 1 | 0 | NA | i; hippocampal sclerosis | Frequent | MFG |  |
| 53 | EOAD | Male | 63 | 10 | 73 | Cachexia/dehydration | 07:00 | 34 | None | A3B3C3 | 5 | 5 | 2 | 5 | 0 | mi’s | Moderate | MFG; + | MFG |
| 54 | LOAD | Female | 75 | 9 | 84 | Cardiac arrest after cachexia | 6:30 | 34 | None | A3B3C3 | ≥4 | 5 | 1 | 0 | NA | None | Sparse | MFG |  |
| 55 | LOAD | Female | 81 | 10 | 91 | Acute abdomen | 10:45 | 34 | None | A3B3C3 | ≥4 | 5 | 1 | 0 | NA | Ischemic foci | Sparse | MFG |  |
| 56 | LOAD | Female | 74 | 15 | 89 | Cardiogenic shock | 6:30 | 34 | None | A3B3C3 | ≥4 | 5 | 1 | 0 | NA | i | Sparse | MFG |  |
| 57 | LOAD | Male | 73 | 9 | 82 | Pneumonia | 04:15 | 34 | None | A3B3C3 | ≥4 | 5 | 2 | 1 | NA | None | None | MFG |  |
| 58 | LOAD | Male | 70 | 10 | 80 | Sudden death | 10:51 | 34 | None | A3B3C3 | 5 | 6 | 1 | 4; Amygdala predominant | ≥2 | Lobar bl; i’s | Sparse | MFG |  |
| 59 | LOAD | Female | 74 | 4 | 78 | Cachexia/dehydration | 07:31 | 34 | None | A3B3C3 | 5 | 5 | 1 | 3 | 0 | mi’s; calcinosis hippocampal vasculature | Sparse | MFG |  |
| 60 | LOAD | Female | 73 | 7 | 80 | Euthanasia | 07:05 | 33 | None | A3B2C2 | 5 | 4 | 1 | 0 | ≥1 | None | None | MFG |  |
| 61 | LOAD | Male | 71 | 13 | 84 | Euthanasia | 05:53 | 34 | None | A3B2C2 | 5 | 4 | 1 | 0 | 0 | None | Sparse | MFG |  |
| 62 | LOAD | Male | 71 | 7 | 78 | Fever eci, cachexia/dehydration | 6:35 | 44 | None | A3B3C3 | ≥4 | 5 | 1 | 0; Amygdala only | NA | None | Moderate | MFG |  |
| 63 | LOAD | Female | 84 | 8 | 92 | Heart failure | 7:00 | 34 | None | A3B3C3 | ≥4 | 5 | 1 | 0 | NA | None | Frequent | MFG |  |
| 64 | LOAD | Female | 85 | 6 | 91 | Cachexia/dehydration | 5:05 | 33 | None | A3B3C3 | ≥4 | 4 | 1 | 0 | NA | None | Frequent | MFG |  |
| 65 | LOAD | Male | 77 | 11 | 88 | Cachexia /dehydration | 05:30 | 34 | None | A3B3C2 | ≥4 | 6 | 1 | 0 | NA | None | Sparse | MFG |  |
| 66 | LOAD | Female | 79 | 6 | 85 | Cachexia/dehydration | 04:05 | 34 | None | A3B3C3 | ≥4 | 5 | 2 | 0 | NA | Ischemic defects | Sparse | MFG |  |
| 67 | LOAD | Male | 78 | 8 | 86 | Cachexia/dehydration | 05:10 | 34 | None | A3B3C1 | 5 | 5 | 1 | 0; Amygdala only | NA | i’s | None | MFG |  |
| 68 | LOAD | Male | 71 | 10 | 81 | Cachexia/dehydration | 07:50 | 34 | None | A3B3C3 | 5 | 6 | 1 | 0 | NA | None | None | MFG |  |
| 69 | LOAD | Female | 80 | 8 | 88 | Cachexia/dehydration | 04:40 | 34 | None | A3B3C3 | 5 | 6 | 1 | 0 | NA | None | None | MFG |  |
| 70 | LOAD | Female | 89 | 7 | 96 | Heart failure and myelodysplastic syndrome | 07:55 | 34 | None | A3B2C2 | 4 | 4 | 1 | 6 | NA | None | Sparse | MFG |  |
| 71 | LOAD | Female | After 65 | NA | 82 | NA | 04:35 | 34 | None | A3B2C1 | 4 | 4 | 0 | 0 | 3 | i; hippocampal sclerosis | None | MFG |  |
| 72 | LOAD | Female | 85 | 5 | 90 | Cerebrovascular accident or gastro-intestinal bleed | 03:55 | 23 | NA | A3B3C3 | 5 | 6 | 0 | 0; Amygdala only | NA | i’s | None | MFG |  |
| 73 | LOAD | Male | 69 | 4 | 73 | NA | 04:45 | 44 | None | A3B3C3 | 4 | 5 | 1 | 0; Amygdala predominant | NA | Hypertensive vasculopathy | Moderate | MFG |  |
| 74 | LOAD | Male | 67 | 10 | 77 | Suicide by overdose | 09:05 | 44 | None | A3B3C3 | 5 | 5 | 1 | 0; Amygdala only | NA | None | Frequent | MFG; + | MFG |
| 75^a^ | CWP | Male | 69 | 8 | 77 | Cardiogenic shock | 05:44 | NA | NA | A3B3C3 | 5 | 5 | 1 | 0 | NA | None |  | MFG |  |
| 76 ^a^ | CWP | Male | 46 | 11 | 57 | Aspiration pneumonia and toxic hepatitis | 05:45 | 33 | NM_000021.3(PSEN1):c.791C>T (p.Pro264Leu) | A3B3C3 | 5 | 6 | 1 | 0 | NA | None |  | MFG |  |
| 77 ^a^ | CWP | Female | 65 | 11 | 76 | Palliative sedation | 06:20 | 34 | None | A3B3C3 | ≥4 | 5 | 1 | 6 | NA | None |  | MFG |  |
| 78 ^a^ | CWP | Male | 64 | 6 | 70 | Cachexia/dehydration | 09:20 | 44 | None | A3B3C3 | 5 | 6 | 1 | 0; Amygdala only | NA | None |  | SPL |  |
| 79 ^a^ | CAA-Type 1 | Female | 86 | 10 | 96 | Cachexia/dehydration | 04:20 | NA | NA | A3B3C2 | ≥4 | 5 | 1 | 0 | NA | None |  | OCC |  |
| 80 ^a^ | CAA-Type 1 | Male | 45 | 19 | 74 | Sudden death | 03:25 | NA | NA | A3B3C3 | ≥4 | 5 | 1 | 0; Amygdala only | NA | None |  | OCC |  |
| 81 ^a^ | CAA-Type 1 | Female | 63 | 12 | 75 | Cachexia/dehydration | 06:00 | NA | NA | A3B3C3 | ≥4 | 5 | 1 | 0 | NA | None |  | OCC |  |

*APOE* genotype and pathogenic mutations are reported when available. ^a^These cases were not included in the semiquantitative scoring for coarse-grained plaques and were only used for comparison with other specific Aβ deposits, being cotton wool plaques and CAA-Type 1 as indicated by the ‘group’ column. Abbreviations: Aβ + amyloid-beta positive cases; bl bleed; CWP cotton wool plaque; EOAD early-onset Alzheimer’s disease; F female; FFFF formalin-fixed free-floating (4%; 24-36 hours); FFPE formalin-fixed paraffin embedded (4%); i infarct; LATE-NC limbic-predominant age-related TDP-43 encephalopathy-neuropathological changes; LB Lewy body; LOAD late-onset Alzheimer’s disease; M male; MFG middle frontal gyrus; mi microinfarct; NA not available; NFT neurofibrillairy tangle; OCC Occipital lobe; PMI post-mortem interval; SPL superior parietal lobe; WM white matter; † death; # number; + temporal -, parietal -, occipital-, olfactory cortex, pre- and post-central gyrus, hippocampus (including CA1-CA4, dentate gyrus, subiculum, and entorhinal cortex), amygdala, caudate nucleus, putamen, substantia nigra, locus coeruleus, pons, medulla oblongata and cerebellum

**Supplementary Table S2** Antibody characteristics and staining details

| Antigen | Antibody | Source | Host | Isotype | Tissue | Method | Dilution | Antigen retrieval^a^ | Incubation | Secondary step^b^ |
| --- | --- | --- | --- | --- | --- | --- | --- | --- | --- | --- |
| Aβ (aa 1-16) | IC-16 | Prof. Dr. Carsten Korth, Heinrich Heine Universität Düsseldorf,  Germany | Mouse | IgG2a | FFPE (5 μm) | IF | 1:200 | HIER in citrate buffer pH 6.0 or tris-EDTA pH 9.0 | 24 hr at RT | Primary directly labeled Alexa 488 |
|  |  |  |  |  | FFFF (60 μm) | IF | 1:200 in 2% BSA/TBS+0,2% Triton | HIER in citrate buffer pH 6.0 or tris-EDTA pH 9.0 | 24 hr at RT | Primary directly labeled Alexa 488 |
| Aβ (aa 8-17) | 6F/3D | Dako | Mouse | IgG1κ | FFPE (6 μm) | IHC | 1:20 | FA 85% (1 hour) + 0.1% trypsin (30 minutes at 37°C) | 24 hr at RT | Biotinylated rabbit anti-mouse (1:200), followed by ABC (1:400) |
| Aβ_40_ (aa 32-40) | MBC40 | Prof. Dr. Haruyasu Yamaguchi, Gunma University School of Health Sciences, Maebashi, Japan | Mouse |  | FFPE (6 μm) | IHC | 1:100 | FA 99% (5 min) | 24 hr at RT | Biotinylated rabbit anti-mouse (1:200), followed by ABC (1:400) |
| Aβ_40_ | G2-10 | Sigma-Aldrich | Mouse | IgG2bκ | FFPE (5 μm) | IF | 1:100 | FA 99% (5 min) | 24 hr at RT | goat-anti-mouse-IgG2 Alexa 488 |
|  |  |  |  |  | FFFF (60 μm) | IF | 1:100 in 2% BSA/TBS+0,2% Triton | FA 99% (10 min) | 24 hr at RT | goat-anti-mouse-IgG2 Alexa 488 |
| Aβ_42_ (aa 37-42) | MBC42 | Prof. Dr. Haruyasu Yamaguchi | Mouse |  | FFPE (6 μm) | IHC | 1:200 | FA 99% (5 min) | 24 hr at RT | Biotinylated rabbit anti-mouse (1:200), followed by ABC (1:400) |
| Aβ_42_ | G2-11 | Sigma-Aldrich | Mouse | IgG1κ | FFPE (5 μm) | IF | 1:100 | FA 99% (5 min) | 24 hr at RT | goat-anti-mouse-IgG1 Alexa 647 |
|  |  |  |  |  | FFFF (60 μm) | IF | 1:100 in 2% BSA/TBS+0,2% Triton | FA 99% (10 min) | 24 hr at RT | goat-anti-mouse-IgG1 Alexa 647 |
| Aβ_N3pE_ | 337.48 | BioLegend | Mouse | IgG1κ | FFPE (6 μm) | IHC | 1:800 | HIER in citrate buffer pH 6.0 | 24 hr at RT | EnVision (Dako) |
| ApoE | E6D7 | Abcam | Mouse | IgG1 | FFPE (6 μm) | IHC | 1:3200 | HIER in citrate buffer pH 6.0 | 24 hr at RT | EnVision (Dako) |
| APP | 3E9 | ThermoFisher | Mouse | IgG1 | FFPE (6 μm) | IHC | 1:6000 | HIER in citrate buffer pH 6.0 | 24 hr at RT | EnVision (Dako) |
| C4b | #AB66791 | Abcam | Rabbit | IgG | FFPE (6 μm) | IHC | 1:1600 | HIER in citrate buffer pH 6.0 | 24 hr at RT | EnVision (Dako) |
|  |  |  |  |  | FFPE (5 μm) | IF | 1:200 | HIER in citrate buffer pH 6.0 | 24 hr at RT | goat-anti-rabbit Alexa 647 |
| CD68 | KP1 | Dako | Mouse | IgG1 | FFPE (5 μm) | IF | 1:300 | HIER in citrate buffer pH 6.0 | 24 hr at RT | goat-anti-mouse Alexa 488 |
|  |  |  |  |  | FFFF (60 μm) | IF | 1:300 in 2% BSA/TBS+0,2% Triton | HIER in citrate buffer pH 6.0 | 48 hr at RT | Biotinylated goat anti-mouse (1:500), followed by ABC (1:400) and tyramide reagent Alexa 594 (1:100 in 0,0015% H_2_O_2_ in TBS) |
| Collagen IV | CIV 22 | Dako | Mouse | IgG1κ | FFPE (6 μm) | IHC | 1:25 | HIER in citrate buffer pH 6.0 | 24 hr at RT | EnVision (Dako) |
| GFAP | 6F2 | Monosan | Mouse | IgG1 | FFPE (6 μm) | IHC | 1:500 | HIER in citrate buffer pH 6.0 | 24 hr at RT | EnVision (Dako) |
| GFAP | #AB5541 | EMD Millipore | Chicken | IgY | FFPE (5 μm) | IF | 1:750 | HIER in citrate buffer pH 6.0 | 24 hr at RT | goat-anti-chicken Alexa 555 |
|  |  |  |  |  | FFFF (60 μm) | IF | 1:750 in 2% BSA/TBS+0,2% Triton | HIER in citrate buffer pH 6.0 | 48 hr at RT | goat-anti-chicken Alexa 555 (in TBS+0,2% Triton) |
| MHC-II | CR3/43 | Dako | Mouse | IgG1 | FFPE (6 μm) | IHC | 1:800 | HIER in citrate buffer pH 6.0 | 24 hr at RT | EnVision (Dako) |
| Laminin | #NB300-144SS | Novus Biologicals | Rabbit | IgG | FFPE (6 μm) | IHC | 1:500 | HIER in citrate buffer pH 6.0 | 24 hr at RT | EnVision (Dako) |
|  |  |  |  |  | FFPE (5 μm) | IF | 1:200 | HIER in tris-EDTA buffer pH 9.0 | 24 hr at RT | EnVision and tyramide reagent Alexa 594 (1:100 in 0,0015% H_2_O_2_ in TBS) |
|  |  |  |  |  | FFFF (60 μm) | IF | 1:500 in 2% BSA/TBS+0,2% Triton | HIER in tris-EDTA buffer pH 9.0 | 24 hr at RT | Biotinylated goat anti-rabbit (1:500), followed by ABC (1:400) and tyramide reagent Alexa 555 (1:100 in 0,0015% H_2_O_2_ in TBS) for 30 min |
| Norrin | #NBP1-59305 | Novus Biologicals | Rabbit | IgG | FFPE (6 μm) | IHC | 1:400 | HIER in citrate buffer pH 6.0 | 24 hr at RT | EnVision (Dako) |
|  |  |  |  |  | FFPE (5 μm) | IF | 1:400 | HIER in tris-EDTA buffer pH 9.0 | 24 hr at RT | goat-anti-rabbit Alexa 647 |
| Norrin (mid-domain) | Custom made | GeneScript | Rabbit |  | FFFF (60 μm) | IF | 1:400 in 2% BSA/TBS+0,2% Triton | HIER in tris-EDTA buffer pH 9.0 | 24 hr at RT | goat-anti-rabbit Alexa 647 |
| p-Aβ at Ser8 | 1E4E11 [31] | Prof. Dr. Jochen Walter, University Hospital Bonn, Bonn, Germany | Mouse | IgG1κ | FFPE (6 μm) | IHC | 1:100 | HIER in citrate buffer pH 6.0 | 24 hr at RT | EnVision (Dako) |
| PrP^C^, PrP^Sc^ | 3F4 | Covance | Mouse | IgG2_A_ | FFPE (6 μm) | IHC | 1:300 | Pre-treatment with FA for 5 min and HIER in citrate buffer pH 6.0  + Proteinase K (Dako) for 5 min for PrP^Sc^ detection | 1 hr at RT | EnVision (Dako) |
| pTau at Ser202 and Thr205 | AT8 | ThermoFisher | Mouse | IgG1 | FFPE (6 μm) | IHC | 1:800 | HIER in citrate buffer pH 6.0 | 24 hr at RT | EnVision (Dako) |

^a^Primary antibodies were diluted in either PBS or normal antibody diluent (ImmunoLogic). ^b^HIER was done by autoclave (FFPE) or by water bath 95 °C for 30 minutes (FFFF). ^c^Secondary antibodies were diluted 1:250 in either PBS or normal antibody diluent (ImmunoLogic) (FFPE) or in TBS (FFFF) unless stated otherwise; secondary antibodies were incubated for 1 hour (FFPE) or 4 hours (FFFF), unless stated otherwise. Abbreviations: aa amino-acid; Aβ amyloid-beta; APP amyloid precursor protein; ApoE apolipoprotein E; FA formic acid; FFFF formalin-fixed (4%; 24-36 hours) free-floating; FFPE formalin-fixed (4%) paraffin-embedded; GFAP glial fibrillary acidic protein; HIER heat-induced epitope retrieval; IF immunofluorescence; IHC immunohistochemistry; ON overnight; PBS phosphate buffered saline; PrP^C^ cellular prion protein; PrP^Scr^ abnormal scrapie isoform of the prion protein; RT room temperature; TBS Tris-buffered saline

**Supplementary Table S3** Overview of coarse-grained plaques scanned using CLSM

Shown is the # of plaques per case that were scanned in z-axis using CLSM per antibody combination (See Table 1 for case details). In a subset of plaques stained for Aβ_40_ / Aβ_42_, an Aβ_40_ shell structure was observed, indicated in the 3^rd^ column. Indicated in the 6^th^ column is the number of coarse-grained plaques that was in direct contact with a vessel

| **Case #** | **# plaques for Aβ_40_ / Aβ_42_** | | **# plaques for Aβ / CD68 / GFAP** | **# plaques for Aβ / norrin / laminin** | |
| --- | --- | --- | --- | --- | --- |
|  |  | **# plaques with Aβ_40_ shell /total plaques** |  |  | **# plaques in direct contact with vessel /total plaques** |
| **29** | 7 | 6/7 | 7 | 9 | 9 / 9 |
| **30** | 6 | 3/6 | 7 | 8 | 5 / 8 |
| **42** | 9 | 5/9 | 7 | 7 | 7 / 7 |
| **53** | 9 | 6/9 | 6 | 11 | 10 / 11 |
| **74** | 7 | 3/7 | 9 | 9 | 6 / 9 |
| Total | 38 | 23/38 (61%) | 36 | 44 | 37 / 44 (84%)^a^ |

^a^Of the 7 plaques not in direct contact with a vessel, only 2 plaques were completely scanned in z-axis. Abbreviations: Aβ Amyloid-beta; Aβ_40_ Amyloid-beta 40; Aβ_42_ Amyloid-beta 42; CLSM Confocal laser scanning microscopy; # number


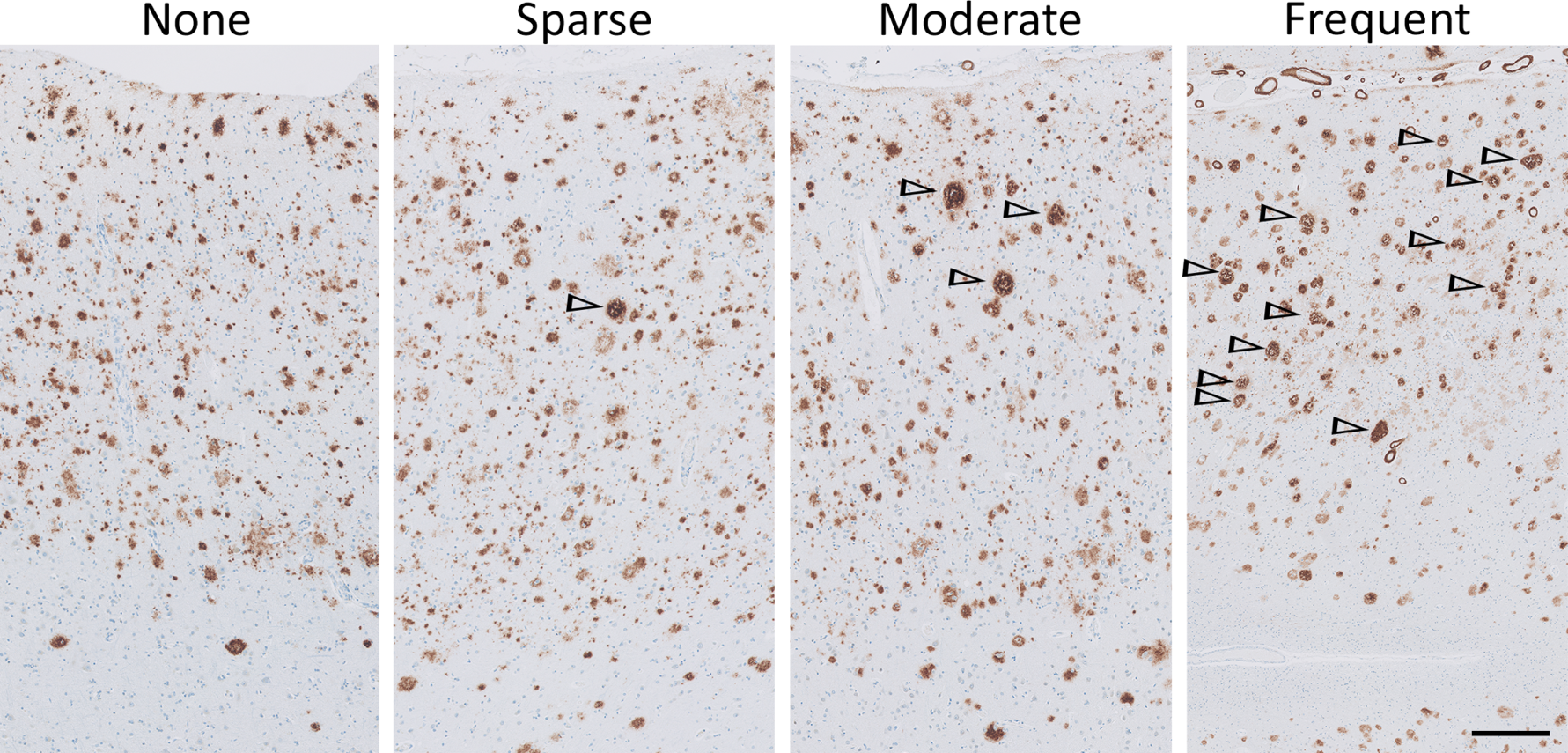


**Supplementary Fig. S4** Semi-quantitative categories for coarse-grained plaque presence in Aβ (6F/3D) immunostaining

Ascending categories of coarse-grained plaque semi-quantitative stages are shown: none; sparse (<6 plaques per 1 cm^2^); moderate (≥6 and ≤30 plaques per 1 cm^2^); and frequent (>30 plaques per 1 cm^2^). Arrowheads indicate coarse-grained plaques. Not all coarse-grained plaques in the last panel are marked due to readability reasons. Scale bar represents 200 µm and is applicable to all images


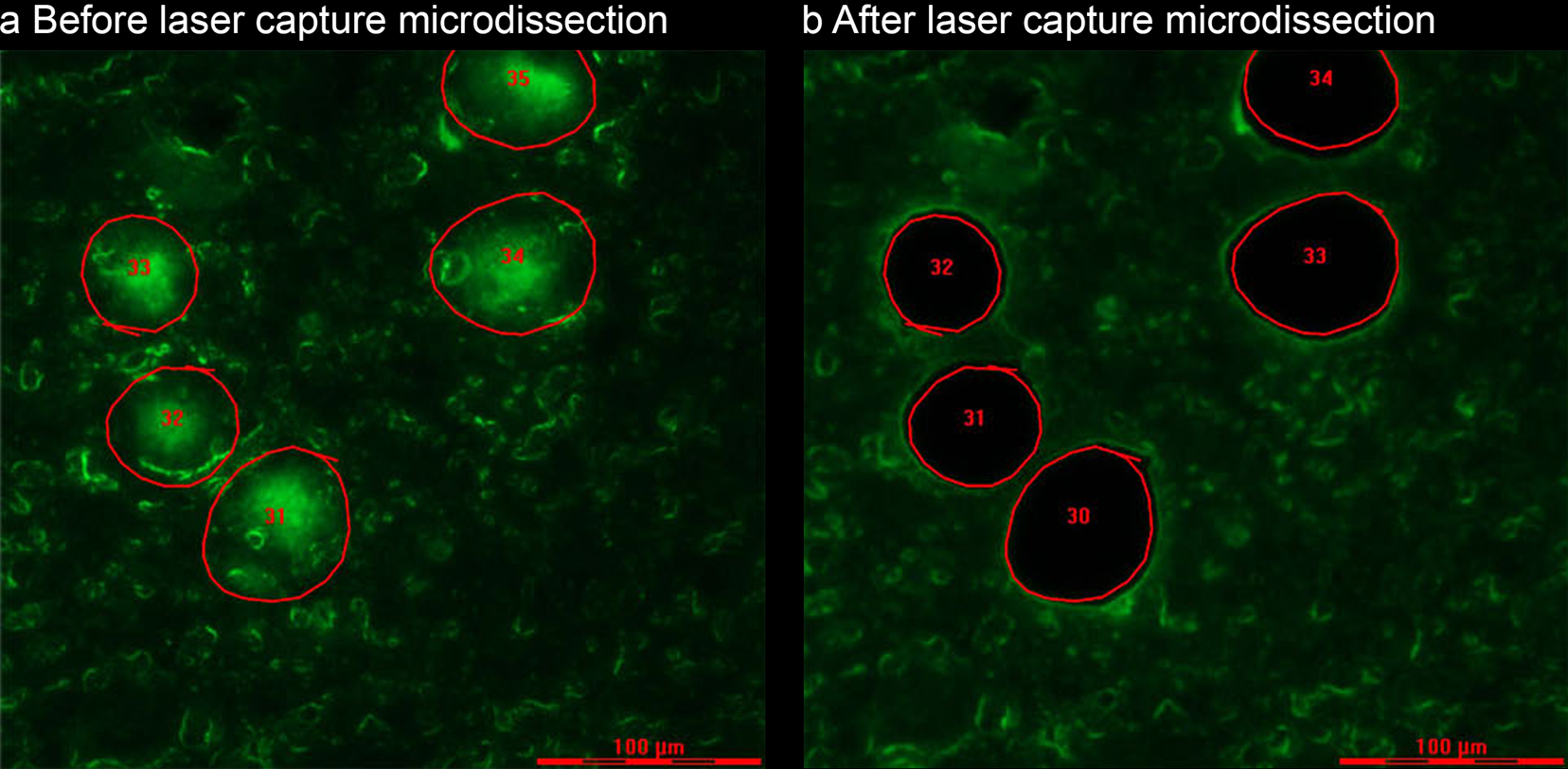


**Supplementary Fig. S5** Visualization of laser-capture microdissection of coarse-grained plaques

**a** Snap-frozen brain sections of case #18 and #32 (Supplementary Table 1 for case details) were stained using thioflavin S. **b** Preparatory to ELISA analysis for Aβ_40_ and Aβ_42_, coarse-grained plaques were laser captured. Abbreviations: Aβ_40_ Amyloid-beta 40; Aβ_42_ Amyloid-beta 42


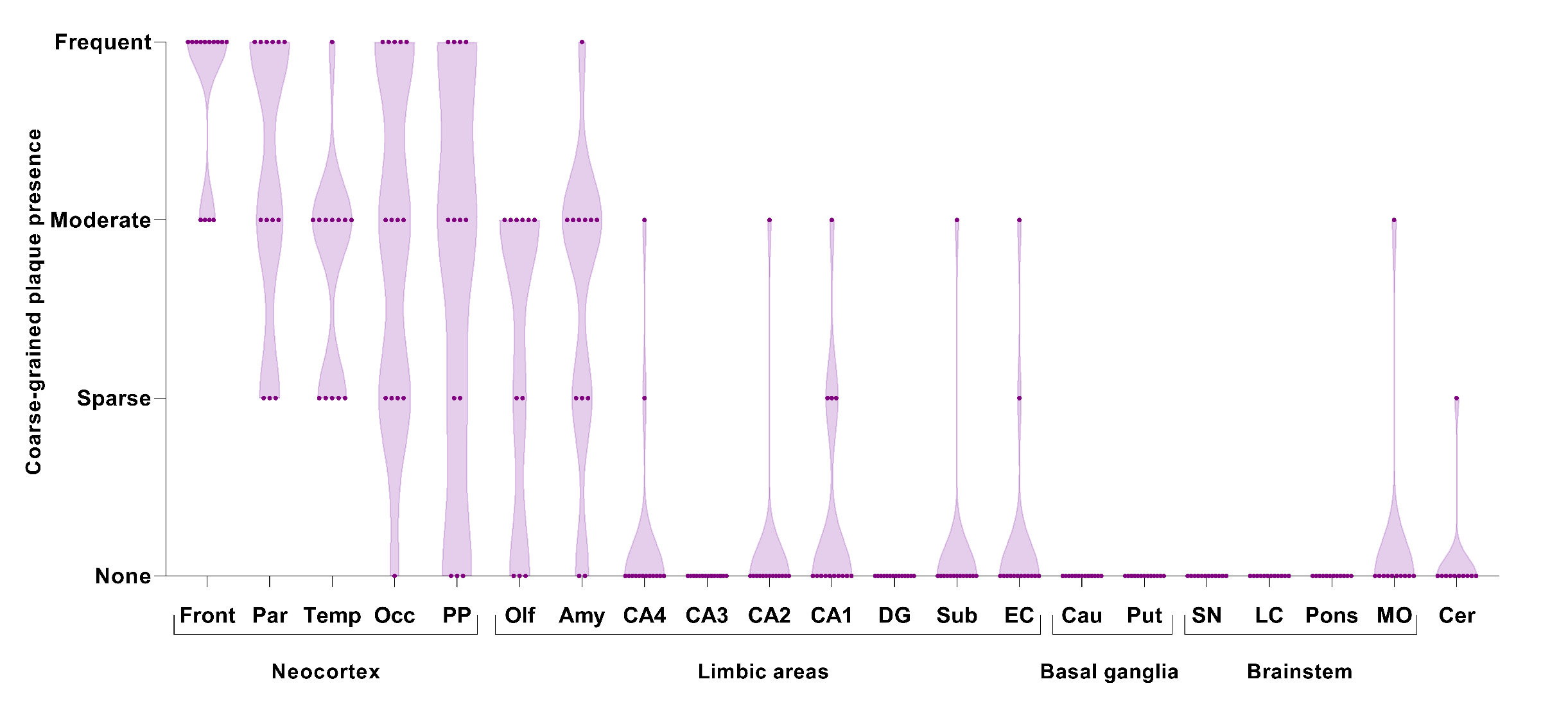


**Supplementary Fig. S6** Distribution of the coarse-grained plaque over different brain regions
The coarse-grained plaque was semi-quantitatively scored (y-axis for scoring categories) in multiple brain regions of 14 cases who had a moderate to frequent score for the coarse-grained plaque in the middle frontal gyrus section. Abbreviations: Amy amygdala; Cau caudate nucleus; Cer cerebellum; DG dentate gyrus; EC entorhinal cortex; Front middle frontal gyrus; LC locus coeruleus; MO medulla oblongata; Occ occipital cortex; Olf olfactory cortex; Par parietal cortex; PP pre-/post central gyrus; SN substantia nigra; Sub subiculum; Temp temporal cortex

**
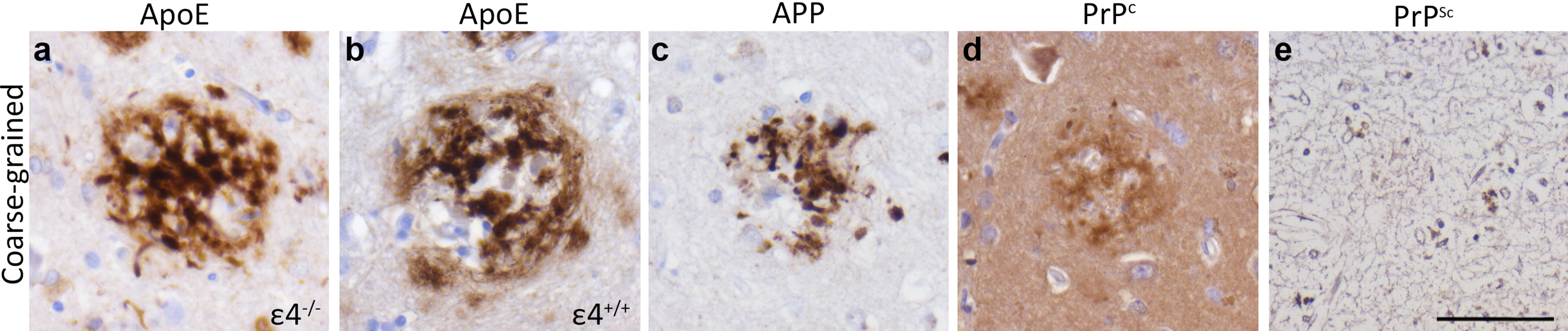
**

**Supplementary Fig. S7** Immunoreactivity for apoE, APP, PrP^C^, and PrP^Sc^ in the coarse-grained plaque
**a**, **b** The coarse-grained plaque was immunoreactive for ApoE in both *APOE* ε4^-/-^ (**a**) and *APOE* ε4^+/+^ (**b**) cases. **c** APP dystrophic neurites were found within the coarse-grained plaque. **d**, **e** The coarse-grained plaque was immunoreactive for PrP^C^ and not for PrP^Sc^. Scale bar represents 50 µm and is applicable to all images. Abbreviations: APP amyloid precursor protein; ApoE apolipoprotein E; PrP^C^ cellular prion protein; PrP^Sc^ scrapie isoform of prion protein


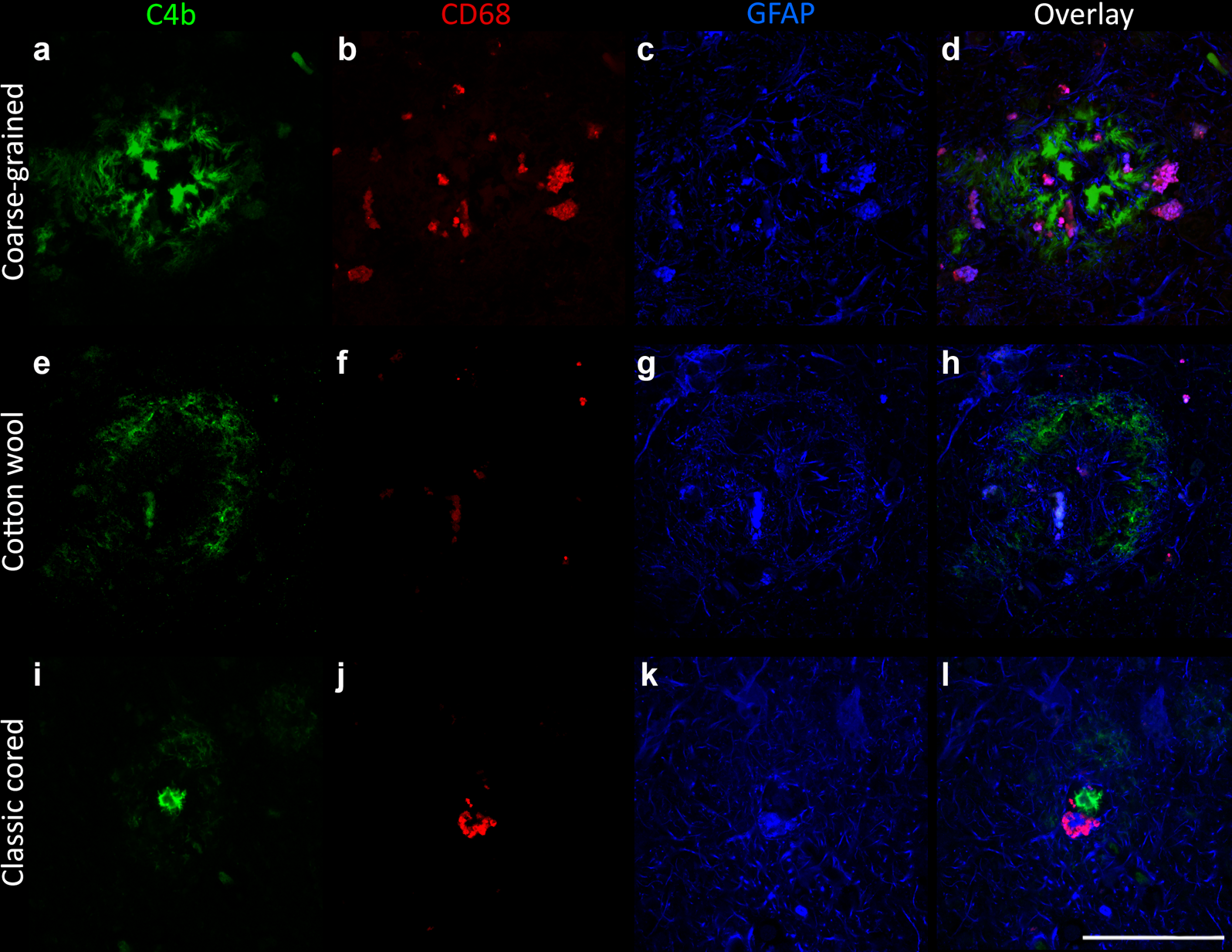


**Supplementary Fig. S8** Triple immunofluorescence for the neuroinflammatory response in the coarse-grained plaque (**a**-**d**) compared to the cotton wool (**e**-**h**) and classic cored plaque (**i-l**). Sections were immunostained using anti-complement factor C4b (green), anti-CD68 (red), and anti-GFAP (blue). The overlay is shown in the last column. Scale bar represents 50 µm and is applicable to all images


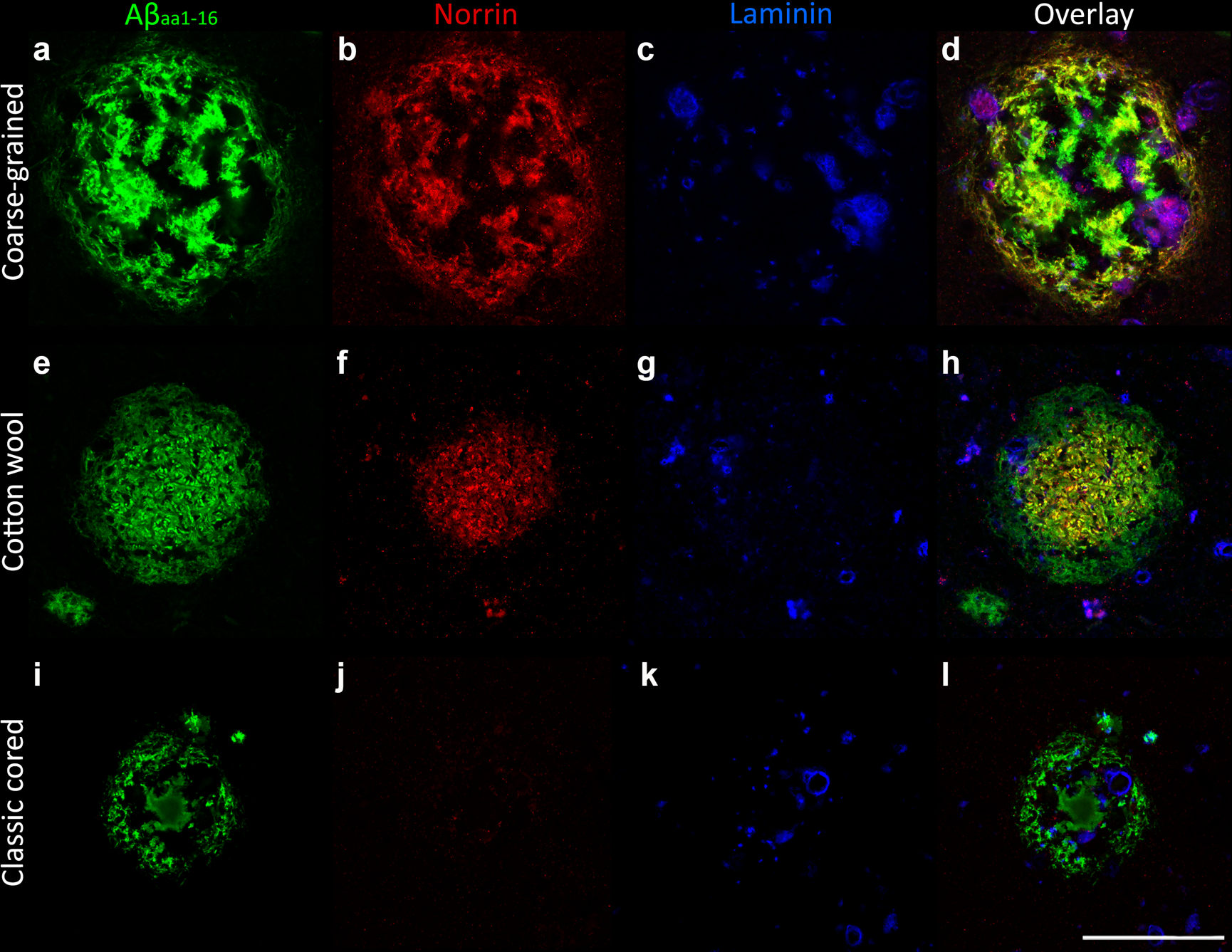


**Supplementary Fig. S9** Triple immunofluorescence for vascular-associated markers in the coarse-grained plaque (**a**-**d**) compared to the cotton wool (**e**-**h**) and classic cored plaque (**i-l**). Sections were immunostained using anti-Aβ aa 1-16 (green), anti-norrin (red), and anti-laminin (blue). The overlay is shown in the last column. Scale bar represents 50 µm and is applicable to all images

**Supplementary Material 2** Aβ_40_ / Aβ_42_ in a coarse-grained plaque in case #30

3D Movie of two coarse-grained plaques stained for Aβ_40_ (green) and Aβ_42_ (red) acquired using confocal laser scanning microscopy. In the smaller coarse-grained plaque Aβ_40_ and Aβ_42_ is co-localized. In the larger coarse-grained plaques Aβ_40_ showed an outer shell structure, surrounding the lesser present Aβ_42_ . Tubular-like structures are visible in Aβ_40_

**Supplementary Material 3** Aβ_40_ / Aβ_42_ in a coarse-grained plaque in case #53

3D Movie of a coarse-grained plaque stained for Aβ_40_ (green) and Aβ_42_ (red) acquired using confocal laser scanning microscopy. The coarse-grained plaque has an Aβ_40_ outer shell structure, surrounding the lesser present Aβ_42_ . Tubular-like structures are visible in Aβ_40_

**Supplementary Material 4** Aβ / CD68 / GFAP in a coarse-grained plaque in case #53

3D Movie of a coarse-grained plaque stained for Aβ (green), GFAP (blue), and CD68 (red) acquired using confocal laser scanning microscopy. GFAP and CD68 signal is seen inside the Aβ-devoid pores of the plaque

**Supplementary Material 5** Aβ / CD68 / GFAP in a coarse-grained plaque in case #29

3D Movie of two coarse-grained plaques stained for Aβ (green), GFAP (blue), and CD68 (red) acquired using confocal laser scanning microscopy. GFAP and CD68 signal is seen inside the Aβ-devoid pores of both plaques

**Supplementary Material 6** Aβ / norrin / laminin in a coarse-grained plaque in case #53

3D Movie of a coarse-grained plaque stained for Aβ (green), laminin (blue), and norrin (red) acquired using confocal laser scanning microscopy. The coarse-grained plaque was in direct contact with a string vessel. A thread of norrin seemed to be connected to the vessel

**Supplementary Material 7** Aβ / norrin / laminin in a coarse-grained plaque in case #29

3D Movie of a coarse-grained plaque stained for Aβ (green), laminin (blue), and norrin (red) acquired using confocal laser scanning microscopy. The coarse-grained plaque was in direct contact with a vessel. A thread of norrin seemed to be connected to the vessel
